# Assessing the efficacy of an integrated intervention to create demand for fishermen’s schistosomiasis and HIV services (FISH) in Mangochi, Malawi: Study protocol for a cluster randomized control trial

**DOI:** 10.1371/journal.pone.0262237

**Published:** 2022-01-07

**Authors:** Donaldson F. Conserve, Sekeleghe Kayuni, Moses K. Kumwenda, Kathryn L. Dovel, Augustine Talumba Choko

**Affiliations:** 1 Department of Prevention and Community Health, Milken Institute of Public Health, George Washington University, Washington, District of Columbia, United States of America; 2 Medical Aid Society of Malawi (MASM), Blantyre, Malawi; 3 Malawi Liverpool Wellcome Trust Clinical Research Programme (MLW), Blantyre, Malawi; 4 David Geffen School of Medicine, The University of California, Los Angeles, California, United States of America; UNITED KINGDOM

## Abstract

**Background:**

Both HIV and schistosomiasis are major public health problems worldwide with 1.8 million new HIV infections, and up to 110 million untreated schistosomiasis cases globally. Although a causal link has not been established, there are strong suggestions that having schistosomiasis increases onward transmission of HIV from co-infected men to women. With both HIV and schistosomiasis treatment readily available in Malawi, there is a need to investigate the feasibility, acceptability and health impacts of joint management of these two hazards, with special focus on health education and demand-creation for fishermen. The aim of this project is to identify optimal models of delivering integrated HIV and schistosomiasis services for fishermen, particularly investigating the effect of using social networks, HIV self-test kits and beach clinic services in Mangochi, Malawi.

**Methods:**

We have mapped 45 boat teams or landing sites for a 3-arm cluster randomized trial using “boat team” as the unit of randomization. The three arms are: 1) Standard of care (SOC) with leaflets explaining the importance of receiving presumptive treatment for schistosomiasis (praziquantel) and HIV services for fishermen, and two intervention arms of 2) SOC + a peer explaining the leaflet to his fellow fishermen in a boat team; and 3) arm 2 with HIV self-test kits delivered to the boat team fishermen by the peer. The primary outcomes measured at 9 months of trial delivery will compare differences between arms in the proportions of boat-team fishermen: 1) who self-report starting antiretroviral therapy or undergoing voluntary medical male circumcision; and 2) who have ≥1 *S*. *haematobium* egg seen on light microscopy of the filtrate from 10mls urine (“egg-positive”).

**Discussion:**

This is the first evaluation of an integrated HIV and schistosomiasis services intervention for fishermen, particularly investigating the effect of using social networks, HIVST kits and beach clinic services. The findings will support future efforts to integrate HIVST with other health services for fishermen in similar settings if found to be efficacious.

**Trial registration:**

This trial is registered in the ISRCTN registry: ISRCTN14354324; date of registration: 05 October 2020. https://www.isrctn.com/ISRCTN14354324?q=ISRCTN14354324&filters=&sort=&offset=1&totalResults=1&page=1&pageSize=10&searchType=basic-search.

Linked to protocol version number 1.4 of 11 January 2021.

## Background

Both HIV and schistosomiasis are major public health problems worldwide with 1.8 million new HIV infections, and up to 110 million untreated schistosomiasis cases globally [1]. HIV prevalence in Malawi is among the highest globally, with 9.6% of the adult population living with HIV, and 39,000 new infections and 17,000 deaths from HIV in 2017 [2]. Fishing communities throughout Africa have much higher HIV prevalence than national averages with, for instance, complex ‘fish-for-sex’ trading networks described in Malawi [3]. In addition, in freshwaters, including Lake Malawi, urinary schistosomiasis (*Schistosoma haematobium*) is a further occupational hazard and, among other pathologies, commonly causes male and female genital lesions resulting in subfertility, haemospermia, genital and pelvic pain [4,5].

Although a causal link has not been established, there are strong suggestions that having schistosomiasis increases onward transmission of HIV from co-infected men to women, and also that schistosomiasis increases susceptibility to HIV in HIV-negative women [4,5]. Most recently, stored serum showed 59% baseline schistosome-antibody positivity in a large Zambian cohort of HIV discordant couples (i.e. only one partner HIV-positive at baseline) that documented 335 HIV seroconversion events during follow-up [5]. Baseline HIV-positive schistosome-positive partners were more likely to transmit HIV to their HIV-negative partner (adjusted hazard ratios 1.8 for men and 1.4 for women), with HIV-negative women who had serological evidence of *S*. *haematobium* at increased risk of acquiring HIV (adjusted hazard ratio 1.4) [5].

International recommendations for high prevalence communities include annual treatment with praziquantel for at-risk adults, such as fishermen, as well as annual mass drug administration (MDA) for school age children [6]. Malawi has delivered several MDA campaigns but mainly to children [7]. A 2018 survey showed poor control in lakeshore men [8]. Engaging adults in MDA programmes will be essential for elimination [9], since schistosomes are long-lived helminth parasites capable of producing eggs for decades if untreated [4]. Egg output depends on the number of mating parasite pairs within the host. Eggs migrate through tissues and are either retained, provoking granuloma formation, or excreted in urine, leading to infection of fresh-water snails [10]. Once treated, acquired immunity results in lasting cure or greatly reduced intensity of reinfection of almost all adults, even if HIV-positive and with ongoing water contact [11] although symptoms may continue due to retained eggs and fibrosis [4].

With both HIV and schistosomiasis treatment readily available in Malawi, there is a need to investigate the feasibility, acceptability and health impacts of joint management of these two hazards, with special focus on health education and demand-creation for fishermen. Remarkable progress has been made towards meeting the 2030 Joint United Nations Programme on HIV/AIDS’ “95-95-95” targets for the HIV care cascade [12]: population-based surveys in 2016 estimated that 72.7% of Malawian people living with HIV (PLHIV) were diagnosed, of whom 88.6% had started antiretroviral treatment (ART), with 90.8% of those virally suppressed [13], which profoundly reduces infectiousness (“treatment-as-prevention”) [3,14–16]. Increased antiretroviral therapy (ART) coverage has reduced annual new infections and deaths in Malawi by an estimated 39% and 50% since 2017 [14]. However, little investment has been made in other HIV prevention services, especially for fishermen due to the mobile nature of their occupation [17]. Similarly, MDA with praziquantel targeting fishermen is not optimally delivered for lack of availability of the fishermen.

Our team and other researchers have successfully reached fishermen with secondary distribution of HIV self-test (HIVST) kits via peers of fishermen to enhance HIV services uptake among this group [18,19]. Secondary distribution of HIVST kits is recommended by the World Health Organization for increasing testing particularly for men who are hard to reach such as fishermen [20]. It is also believed that having schistosomiasis may increase the risk of HIV transmission and acquisition in men. Such a risk, although not yet established albeit with biological plausibility [10], implies that there is urgent need to find optimal models of increasing demand for HIV and schistosomiasis services among fishermen. Thus, the project builds on the previous experience with reaching fishermen and aims to evaluate peer-based approaches of increasing demand for HIV and schistosomiasis services among fishermen in Mangochi with or without the offer of HIVST kits.

## Methods/Design

### Study design and setting

**Fig 1:**
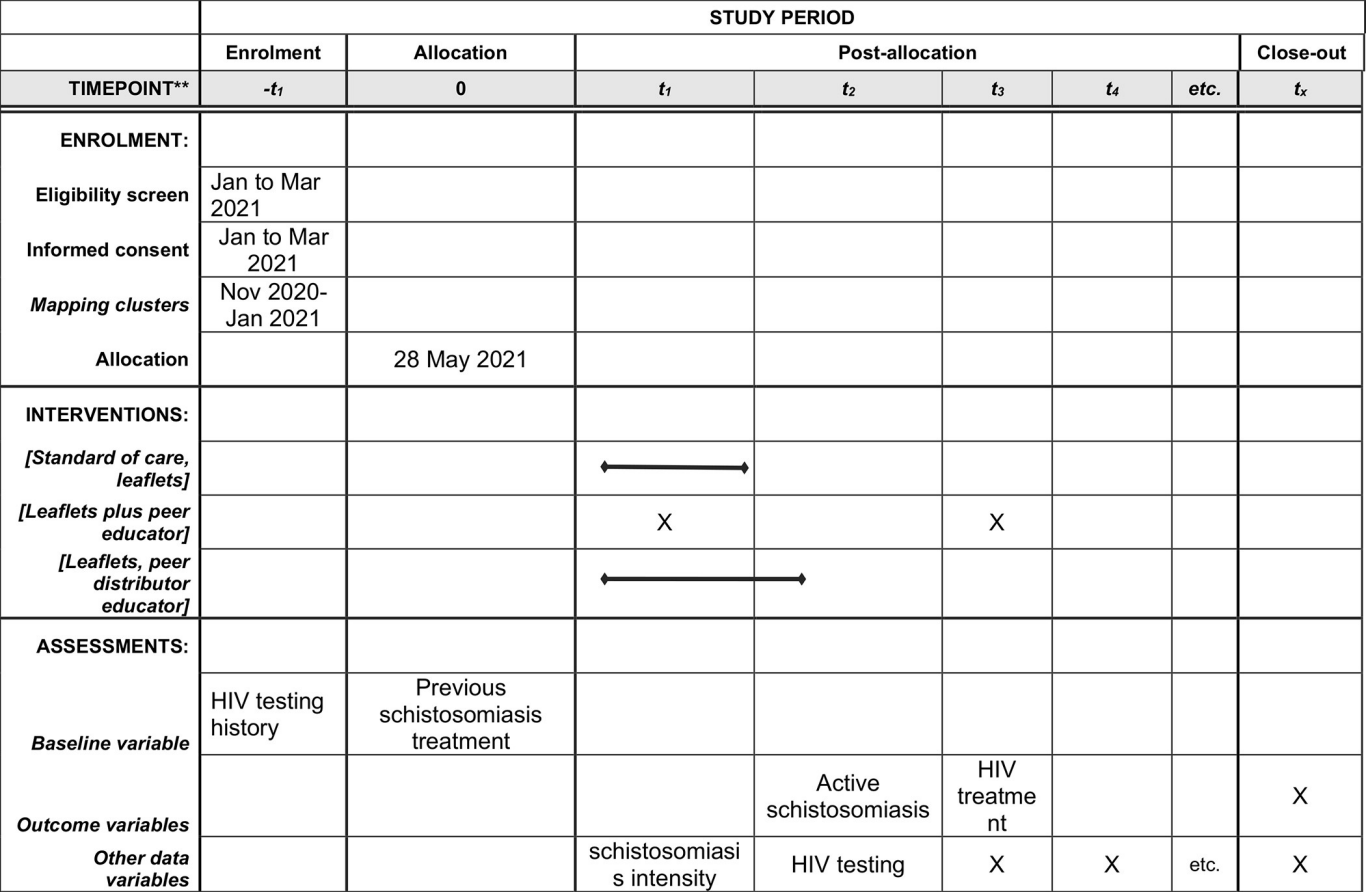
SPIRIT figure summarizing trial schedule.

**Fig 2:**
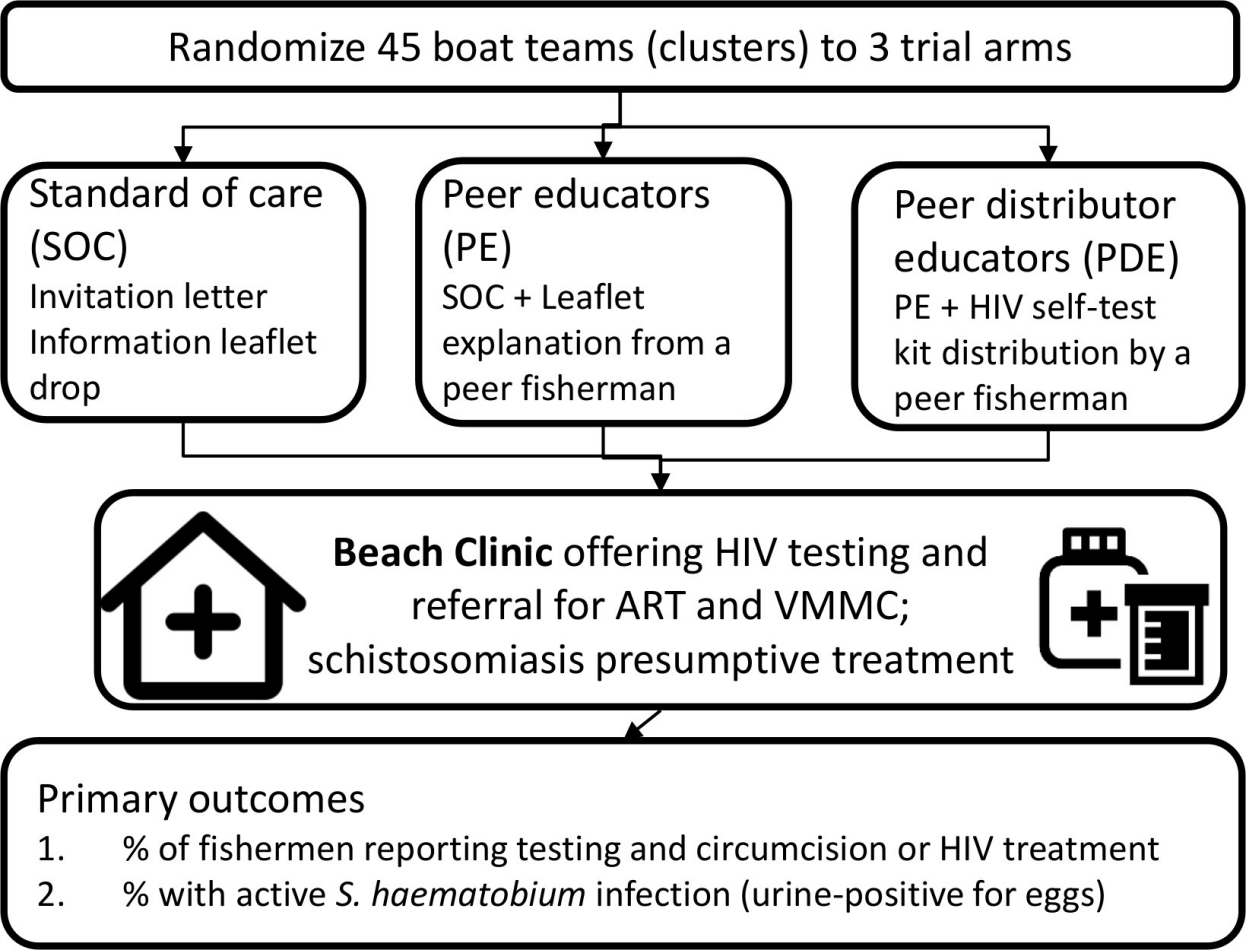
Trial schema.

This is a 3-arm cluster randomized trial (CRT) using “boat team” as the unit of randomization with participant enrollment started in May 2021 (Fig 1). A “boat team” is defined as a landing site or part of land from which fishermen dock. The intervention arms will compare 3 strategies (Fig 2) for creating demand for services among fishermen. Mangochi district, where the CRT will take place, is in the eastern region of lake Malawi bordering Mozambique in the southern part of Lake Malawi and has an adult (15–49 years) HIV prevalence of 10.1% [21].

### Mapping and enumeration

**Fig 3:**
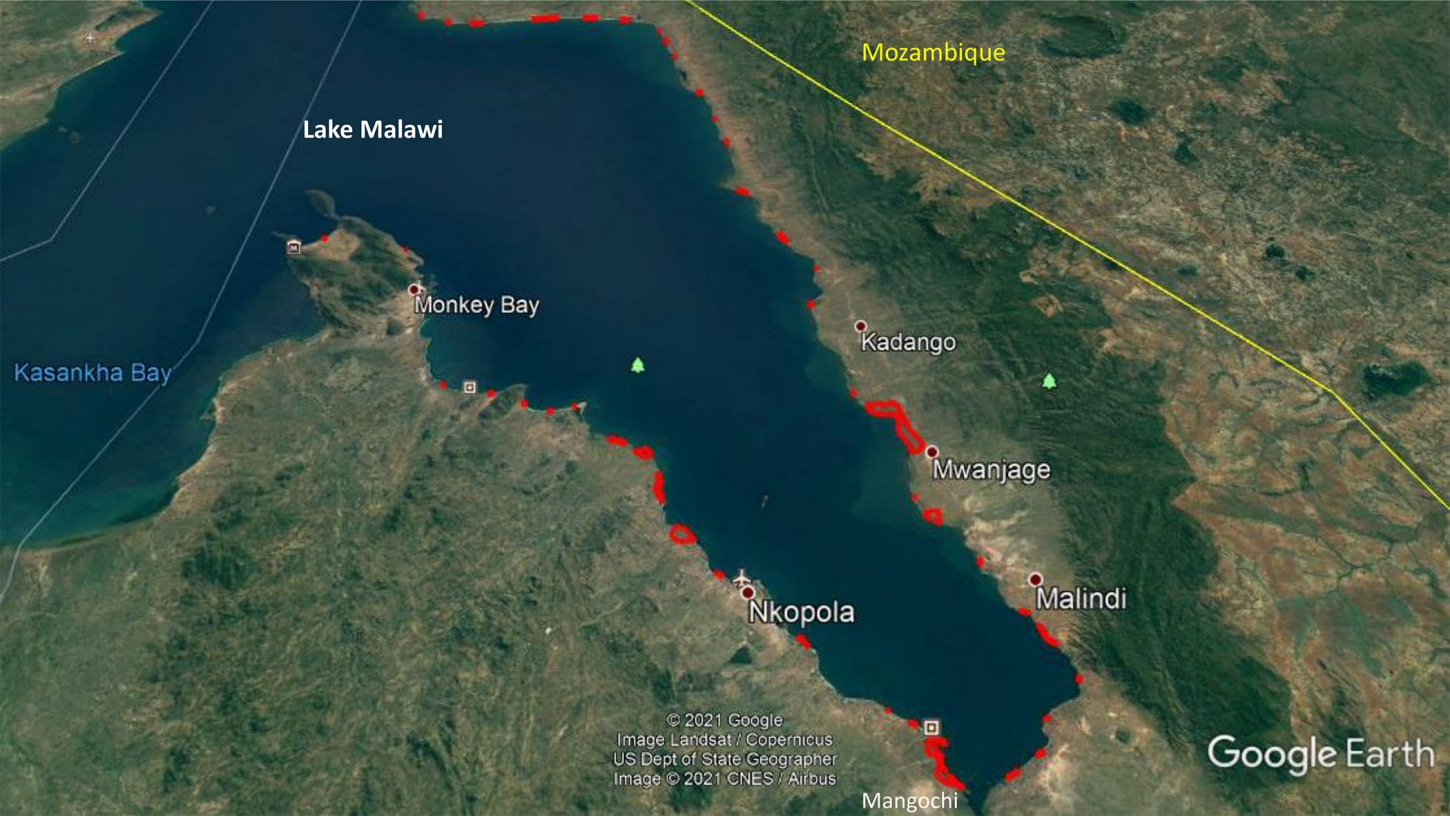
Map of Mangochi district in Malawi showing study clusters. Source: Google Earth Pro with study clusters drawn by the study team.

**Fig 4:**
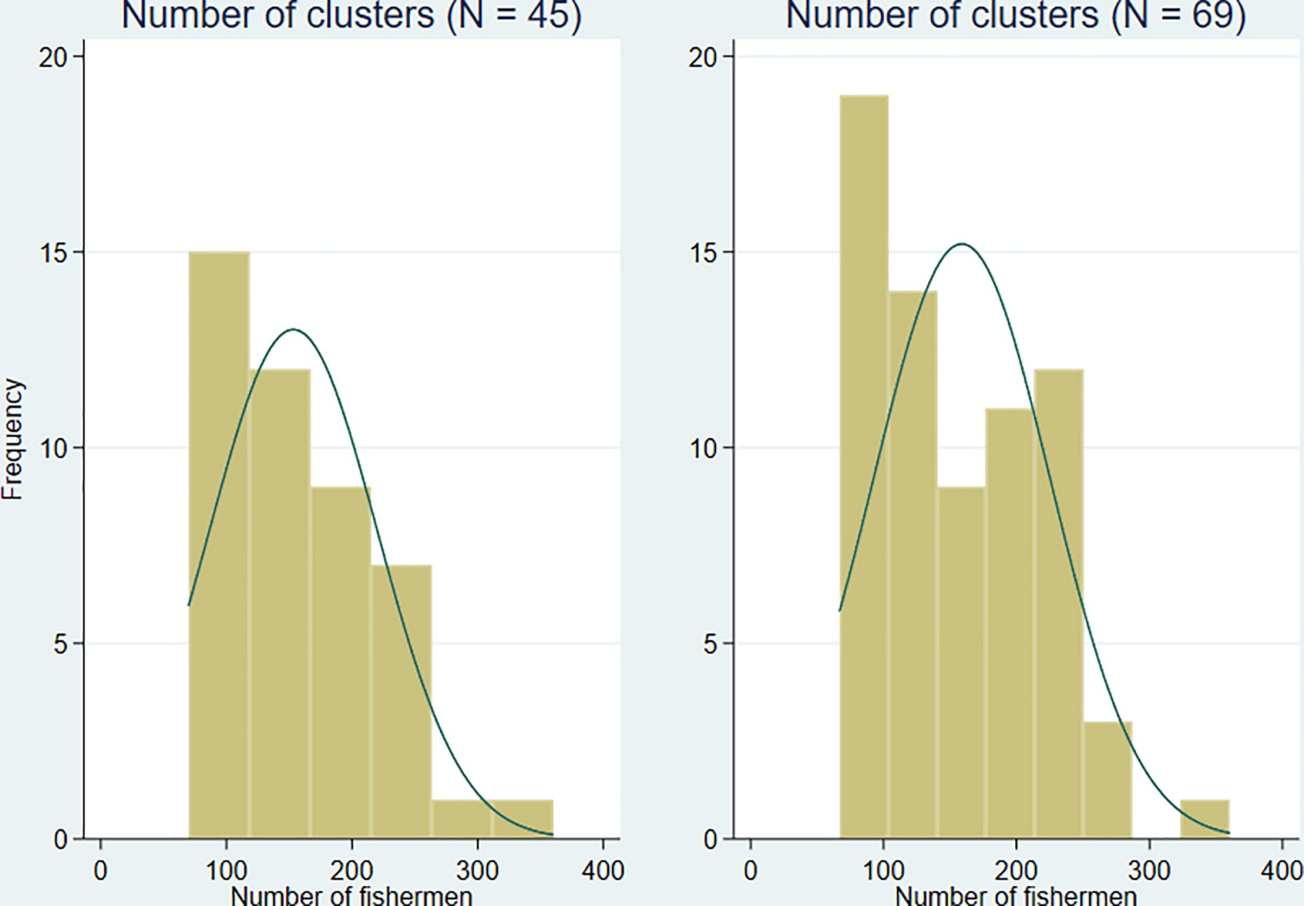
Distribution of number of fishermen in study clusters.

The initial activities, which occurred between November 2020 to January 2021, involved mapping the boat teams or landing sites through circumferential walk with global positioning system devices (Fig 3). The aim was to demarcate landing sites comprising ~100 fishermen. The exercise also created “buffer” zones (i.e. sufficient gaps between any two landing sites to avoid contamination during trial delivery) (Fig 3). Potential locations for beach clinics were also identified and marked during this exercise. Overall, 69 clusters were identified and mapped with an overall harmonic mean number of 132.5 (95% confidence interval [CI]: 119.9; 148.0); range 67 to 360. Number of fishermen in clusters was positively skewed (Fig 4).

To arrive at the 45 clusters required by the trial the following pre-specified exclusion criteria were applied: 1) Any two clusters must be at least 500 meters apart as a measure to prevent potential contamination; 2) For clusters in violation of rule 1: toss of a coin to decide which of the two clusters to include in the set of 45; 3) The harmonic mean number of fishermen for the chosen set of 45 clusters should be at least 100 fishermen; 4) If rule 3 is violated, start again and include larger clusters not withstanding rule 1 i.e. choose the larger between two clusters that are less than 500 meters apart; 5) For any three clusters on a colinear path with distance less than 500 meters between any given two clusters will lead to the middle cluster being dropped; and 6) Having applied rules 1–5 yet the number of clusters remains more than 45, clusters that are furthest will be removed one at a time from either end until only 45 clusters remain that satisfied rule 3.

The result of applying the above criteria was the removal of 24 clusters the majority of which had less than 100 fishermen. The harmonic mean number for the remaining 45 clusters was 128.0 (95% CI: 113.5; 146.6); range 70 to 360 and the distribution of number of fishermen remained positively skewed (Fig 3). Following the mapping exercise, a survey was conducted with fishermen in the final 45 selected landing sites with questions including socio-demographic data, some schistosomiasis-related questions and questions around willingness to attend the beach clinic. The main inclusion criterion was a male resident fisherman with exclusions on the basis of being younger than 18 years old, non-resident fisherman.

### Trial hypothesis

The study will test the hypothesis that HIVST promoted by peer educators (PE) or provided by peer-distributor-educators (PDE) will achieve higher coverage of health interventions (recent HIV testing, linkage to voluntary male medical circumcision (VMMC) and ART as indicated, and praziquantel) than beach-side services alone, and will leave men with increased understanding of the benefits of early treatment and prevention for both diseases.

### Recruitment and randomization

Boat teams were randomised 1:1:1 by an independent statistician based at London School of Hygiene & Tropical Medicine using computerized restricted block randomization (geographical spread, cluster size, traditional authority, and HIV and schistosomiasis estimates) with final arm allocation assigned at a public randomisation ceremony held on 28 May 2021. Investigator masking was maintained for technical but not field staff or participants. It was not practical to blind either the participants or the investigator in this study because of the nature of the interventions which includes collection of different study materials such as HIVST kits. However, all data will be managed without reference to the study arm until the final data analysis, thus providing masking to main investigators and collaborators.

### Beach clinic

A beach clinic, providing HIV testing, confirmatory testing and referral for ART and voluntary male medical circumcision will be set up as part of the trial. The beach clinic will also provide presumptive praziquantel to all attendees. Beach clinic services will be provided from two tents located strategically to cover five clusters per round. Each beach clinic will be manned by experienced staff: one nurse-preferably seconded from Mangochi District Hospital; one HIV diagnostic assistant and one laboratory microscopist. The administration of praziquantel will have clinical oversight by a Doctor (co-investigator).

### Trial arms and interventions

In the standard of care arm (SOC), demand for beach clinic services will be through mere provision of leaflets in the SOC arm via selected fishermen. For boat-teams randomized to peer-intervention arms, 2 to 5 PEs and PDEs were peer-nominated per boat-team for training by the study team in basic HIV and schistosomiasis facts, and in how to pass on key messages to others using educational materials provided. In the PE arm: PEs will actively explain the trial leaflet and encourage fellow fishermen to attend the beach clinic with a pre-allocated invitation letter. In the PDE arm: PDEs will follow PE arm procedures plus the provision of HIVST kits to their fellow boat crews. PDEs will be trained in HIVST for secondary distribution. Information and educational materials demonstrating correct use of HIVST kits will be part of the training. PDE/PEs will receive US$20 on meeting agreed intervention targets. PEs will receive HIV/schistosomiasis educational and promotion materials and barcoded colored vouchers to distribute to boat-team members (5 vouchers per client) promoting Beach Clinic service uptake. Voucher recipients will be recorded in a register. PDEs will receive educational materials as above and a starter supply of 50 HIVST kits for distribution, recorded in an HIVST register.

### Primary and secondary outcomes

Primary outcomes measured at 9 months of trial delivery will compare differences between arms in the proportions of boat-team fishermen: 1) who self-report starting ART or undergoing VMMC; and 2) who have ≥1 *S*. *haematobium* egg seen on light microscopy of the filtrate from 10mls urine (“egg-positive”). Secondary outcomes will compare differences between arms in: 1) self-reported recent (last 9 months) HIV testing; 2) self-reported HIV prevention knowledge score; 3) self-reported schistosomiasis knowledge score; 4) self-reported high-risk sex in the last month; and 5) *S*. *haematobium* intensity. Arms will also be compared for difference in self-reported praziquantel uptake and intensity of infection using egg count per 10mls of urine. Uptake of HIV and praziquantel in broader social networks will be investigated by constructing egocentric social networks.

### Outcome measurements

28 days after the last round of beach clinic services, an endline survey will follow up all consenting (S1 File) boat-team members (~4,500 individuals) through the peer leaders. Fishermen will be requested to attend the beach clinic for a face-to-face interview, finger prick blood for immediate HIV testing, and a urine sample for egg-microscopy. Data will be extracted from the Beach Clinic registers, using barcode readers to allocate trial arm origin of each participant.

All solicited and unsolicited adverse events will be reported according to the local institutional review board (IRB) guidelines and forms. Participants who experience harms related to the study will be cared for by linking them to appropriate services.

### Sample size and statistical analyses

Using established cluster-randomized methodology [22] 15 boat-teams/arm (1,500 fishermen/arm) will provide 80% power to detect a 9% increase in combined ART/VMMC uptake compared to an assumed 10% under SOC [23]. We assume intercluster coefficient of variation (*k*) of 0.20, with HIVST uptake of 50%-80% [18,23] (Table 1). For the second primary outcome, we assume praziquantel uptake will be 10% to 20% higher in PDE/PE arms than an assumed 40–60% for the SOC arm, with baseline egg-positivity 15–25%, 95% cure from praziquantel, and *k* 0.20–0.30 [7,24]. Power provided by the 15 clusters/arm for HIV endpoints is >80% over most of this range of scenarios.

**Table 1:** Assumptions and parameters for sample size.

**Assumptions**	
Average cluster size (number of men in a fishing dock)	100
Proportion eligible for the trial (mainly not already on ART)	0.80
Proportion accepting to self-test	0.50–0.80[18,23]
Proportion HIV positive or HIV negative and uncircumcised	0.20–0.50[23]
Uptake of praziquantel in SOC arm by endline	0.4–0.6
Proportion urine egg-positive in SOC arm (endline)	0.09–0.17
Geometric mean egg-count if egg-positive in SOC (endline)	10/10mL
**Parameters**	
Significance level (α)	0.05
Power (1-β)	0.80
Allocation ratio	1:1:1

Trial analyses will be conducted with R [25] and Stata 14.0 and will follow the statistical analysis plan (S2 File). Baseline and endline characteristics will be computed as proportions or median (interquartile range [IQR]), or geometric mean (eggs/10ml), as appropriate, by arm. Imbalances will be adjusted for in primary and secondary outcomes. A test of null hypothesis of no difference in effectiveness of each intervention compared to SOC will be conducted. All analyses will be done by intention to treat. The clustered design will be taken into account during analysis. In each cluster, the proportion of fishermen achieving the primary outcome (s) will be calculated.

$$\frac{number\;of\;fishermen\;achieving\;the\;primary\;outcome\;(s)}{number\;of\;fishemen\;eligible}$$

Cluster level summaries will be examined graphically in each trial arm to determine the distribution of the cluster proportion with the primary outcome (s) [22]. If the distributions are skewed, logarithm will be applied to approximate normal distributions. The mean of proportions in each of the two intervention arms will be compared to the SOC arm using unpaired t-test. A risk ratio and 95% confidence interval (CI) will be computed for each comparison by dividing the mean of proportions in each intervention arm and the mean of proportions in the SOC arm. A random effects logistic regression model will also be fitted to the individual level data to estimate the odds ratio and 95% CI for each of the intervention arms. These models will also be used to estimate the coefficient of variation (*k*) for each of the two primary outcomes.

The analysis for all secondary outcomes that are measured as a proportion will proceed in a similar fashion to the analysis of the primary outcomes as described for the primary outcomes. For the HIV and schistosomiasis knowledge outcomes, a binary outcome coded as 1 = for those showing knowledge and 0 = for those without adequate knowledge will be first generated based on the median knowledge score. The secondary outcome of *S*. *haematobium* intensity will begin with a histogram plotted to examine the distribution of eggs per 10ml of urine. The geometric mean and standard deviation of eggs per 10ml urine will then be computed for each trial arm. An incidence rate ratio and 95% CI will be computed by fitting a negative binomial model.

No data monitoring board will be set up in this study as no interim analysis is planned. However, a trial steering committee will provide oversight for the study. Independent trial audits will also be conducted by the local IRB. Any important changes to the study protocol (S3 File) will be communicated by the principal investigator to all stakeholders in accordance with IRB requirements.

## Discussion

To the best of our knowledge, this is the first evaluation of an integrated HIV and schistosomiasis services intervention for fishermen, particularly investigating the effect of using social networks, HIVST kits and beach clinic services. The Rift Valley in Africa suffers high prevalence of both HIV and schistosomiasis, and although the pathology is different, HIV and *Schistosoma* haematobium have important commonalities. First, both are hazards for fishermen with diagnosis being a major barrier to treatment [8,17]. Secondly, recent studies have suggested that female genital schistosomiasis increases the risk of HIV infection 3 to 4 times in women and although the link between HIV and genital schistosomiasis is not yet established in men there is biological plausibility [5]. Thirdly, controlling HIV in fishing communities may also require tackling schistosomiasis because while policies are in place their implementation is suboptimal [10,17]. The manuscript is summarized through the SPIRIT Checklist (S4 File).

As elsewhere, multiple barriers prevent and delay Malawian men from seeking health-seeking relative to women, with lower coverage of lifetime HIV testing (69.9% vs 83.3% in women [13,26] and more advanced HIV at ART initiation [21]. Harmful masculine norms relating to health service usage [27–30] in part explain this, but men also face substantial access barriers especially when trying to access clinic services from a position of job insecurity [31,32]. Clinics tend to be structured around maternal and child health in a way that unintentionally alienates and disadvantages men [31,32]. Therefore, uptake of effective HIV prevention approaches remains low with 72.2% of men being uncircumcised, and condom use is suboptimal even with non-primary/transactional partners [2]. In addition, fishermen face multiple risk in their jobs that may be associated with sexual risk taking and increase their HIV risk. From the male perspective, fishing jobs with the highest risk of drowning typically pay a share of the catch to low-status migrant labourers, for whom risk-taking behaviour with alcohol and transactional sex can then provide temporary relief from the dangerous, physical demands and insecurity of their work [3]. For women, transactional sex can bring favourable access to quality fish and a way to recover lost capital from unsold spoilt fish [3].

Given the barriers and risk fishermen face and their mobility, an integrated approach to provide HIV and schistosomiasis services can improve efficiency and address multiple health topics for this group. Evidence from integrated services for HIV and other health topics have found them to be cost-effective [33]. The project will also be one of the first to integrate HIVST with another health service. The majority of the HIVST studies and programs targeting men have been implemented as HIVST alone [34]. The findings will support future efforts to integrate HIVST with other health services for men. Given the high uptake of HIVST among men, there is potential to reach men for other health services integrated with HIVST. For example, our previous experience with reaching male partners of pregnant women with HIVST has demonstrated that this strategy is effective. Partner-delivered HIVST showed HIVST to be strongly preferred over other testing modalities, with high uptake (87.0% to 95.4%) of men self-testing using kits delivered by their pregnant partner [23,35,36]. In addition, our recent pilot study in Uganda showed high acceptability (82% of 116 men offered a kit accepting to self-test) and safety of peer-delivered HIVST to fishermen [18].

The existing evidence of high HIVST uptake among men through different intervention strategies support the potential for integrating HIVST with other health services. Thus, an integrated community-led MDA and peer-based interventions can also reach remote or hard-to-reach populations such as fishermen more effectively than standard interventions [37]. Malawi National Policy already supports praziquantel presumptive treatment and peer-based HIVST delivery for male workplaces [38]. Delivering HIVST along with additional interventions can encourage prompt uptake of VMMC and ART, and schistosomiasis treatment [23,39].

## Supporting information

S1 File Model consent form. (DOC)

S2 File Statistical analysis plan. (DOCX)

S3 File Protocol. (DOCX)

S4 File SPIRIT checklist. (DOC)

## Abbreviations

SOC: standard of care
HIVST: HIV self-testing
MDA: mass drug administration
ART: antiretroviral therapy
CRT: cluster randomized trial (CRT)
PE: peer educator
PDE: peer-distributor-educators
VMMC: voluntary male medical circumcision
CI: confidence interval

## References

[1] Léger E, Borlase A, Fall CB, Diouf ND, Diop SD, Yasenev L, et al. Prevalence and distribution of schistosomiasis in human, livestock, and snail populations in northern Senegal: a One Health epidemiological study of a multi-host system. The Lancet Planetary health. 2020;4(8):e330–e42. Epub 2020/08/18. doi: 10.1016/S2542-5196(20)30129-7 ; PubMed Central PMCID: PMC7443702. 32800151 PMC7443702

[2] Joint United Nations Programme on HIV and AIDS (UNAIDS). UNAIDS Data. Geneva, Switzerland: UNAIDS, 2018.

[3] MacPherson EE, Sadalaki J, Njoloma M, Nyongopa V, Nkhwazi L, Mwapasa V, et al. Transactional sex and HIV: understanding the gendered structural drivers of HIV in fishing communities in Southern Malawi. Journal of the International AIDS Society. 2012;15 Suppl 1:1–9. doi: 10.7448/IAS.15.3.17364 ; PubMed Central PMCID: PMC3499929. 22713352 PMC3499929

[4] Kayuni S, Lampiao F, Makaula P, Juziwelo L, Lacourse EJ, Reinhard-Rupp J, et al. A systematic review with epidemiological update of male genital schistosomiasis (MGS): A call for integrated case management across the health system in sub-Saharan Africa. Parasite epidemiology and control. 2019;4:e00077. Epub 2019/01/22. doi: 10.1016/j.parepi.2018.e00077 ; PubMed Central PMCID: PMC6324017. 30662962 PMC6324017

[5] Wall KM, Kilembe W, Vwalika B, Dinh C, Livingston P, Lee YM, et al. Schistosomiasis is associated with incident HIV transmission and death in Zambia. PLoS neglected tropical diseases. 2018;12(12):e0006902. Epub 2018/12/14. doi: 10.1371/journal.pntd.0006902 ; PubMed Central PMCID: PMC6292564. 30543654 PMC6292564

[6] World Health Organization. Accelerating work to overcome the global impact of neglected tropical diseases: a roadmap for implementation. Geneva, Switzerland: WHO, 2011.

[7] Kayuni S, Peeling R, Makaula P. Prevalence and distribution of Schistosoma haematobium infection among school children living in southwestern shores of Lake Malawi. Malawi medical journal: the journal of Medical Association of Malawi. 2017;29(1):16–23. Epub 2017/06/02. doi: 10.4314/mmj.v29i1.4 ; PubMed Central PMCID: PMC5442486. 28567191 PMC5442486

[8] Witek-McManus S, Simwanza J, Chisambi AB, Kepha S, Kamwendo Z, Mbwinja A, et al. Epidemiology of soil-transmitted helminths following sustained implementation of routine preventive chemotherapy: Demographics and baseline results of a cluster randomised trial in southern Malawi. PLoS neglected tropical diseases. 2021;15(5):e0009292. Epub 2021/05/13. doi: 10.1371/journal.pntd.0009292 . 33979325 PMC8224978

[9] Toor J, Alsallaq R, Truscott JE, Turner HC, Werkman M, Gurarie D, et al. Are We on Our Way to Achieving the 2020 Goals for Schistosomiasis Morbidity Control Using Current World Health Organization Guidelines? Clinical infectious diseases: an official publication of the Infectious Diseases Society of America. 2018;66(suppl_4):S245–s52. Epub 2018/06/04. doi: 10.1093/cid/ciy001 ; PubMed Central PMCID: PMC5982704. 29860290 PMC5982704

[10] Bustinduy A L, King C H. Schistosomiasis. In: Farrar J, Hotez P, Junghanss T, Lalloo D, White N, editors. Manson’s Tropical Diseases. 23 ed. London: Elsevier; 2014.

[11] Mwanakasale V, Vounatsou P, Sukwa TY, Ziba M, Ernest A, Tanner M. Interactions between Schistosoma haematobium and human immunodeficiency virus type 1: the effects of coinfection on treatment outcomes in rural Zambia. The American journal of tropical medicine and hygiene. 2003;69(4):420–8. Epub 2003/12/03. . 14640503

[12] Joint United Nations Programme on HIV and AIDS (UNAIDS). FAST-TRACK: Ending the AIDS epidemic by 2030. Geneva, Switzerland: UNAIDS, 2014.

[13] Malawi Ministry of Health. Malawi Population-based HIV Impact Assessment (MPHIA) 2015–16: First Report. Lilongwe, Malawi: 2017.

[14] Joint United Nations Programme on HIV and AIDS (UNAIDS). Ending AIDS. Progress towards the 90–90–90 targets. Geneva, Switzerland: UNAIDS, 2017.

[15] Cohen MS, Chen YQ, McCauley M, Gamble T, Hosseinipour MC, Kumarasamy N, et al. Antiretroviral Therapy for the Prevention of HIV-1 Transmission. The New England journal of medicine. 2016;375(9):830–9. doi: 10.1056/NEJMoa1600693 ; PubMed Central PMCID: PMC5049503. 27424812 PMC5049503

[16] Rodger AJ, Cambiano V, Bruun T, Vernazza P, Collins S, van Lunzen J, et al. Sexual Activity Without Condoms and Risk of HIV Transmission in Serodifferent Couples When the HIV-Positive Partner Is Using Suppressive Antiretroviral Therapy. Jama. 2016;316(2):171–81. Epub 2016/07/13. doi: 10.1001/jama.2016.5148 . 27404185

[17] Toms K, Potter H, Balaba M, Parkes-Ratanshi R. Efficacy of HIV interventions in African fishing communities: A systematic review and qualitative synthesis. International journal of infectious diseases: IJID: official publication of the International Society for Infectious Diseases. 2020;101:326–33. Epub 2020/10/06. doi: 10.1016/j.ijid.2020.09.1476 . 33017696

[18] Choko AT, Nanfuka M, Birungi J, Taasi G, Kisembo P, Helleringer S. A pilot trial of the peer-based distribution of HIV self-test kits among fishermen in Bulisa, Uganda. PloS one. 2018;13(11):e0208191. doi: 10.1371/journal.pone.0208191 . 30496260 PMC6264512

[19] Matovu JKB, Bogart LM, Nakabugo J, Kagaayi J, Serwadda D, Wanyenze RK, et al. Feasibility and acceptability of a pilot, peer-led HIV self-testing intervention in a hyperendemic fishing community in rural Uganda. PLoS One. 2020;15(8):e0236141. Epub 2020/08/09. doi: 10.1371/journal.pone.0236141 ; PubMed Central PMCID: PMC7413506. 32764751 PMC7413506

[20] WHO. Guidelines on HIV self-testing and partner notification: supplement to consolidated guidelines on HIV testing services. Geneva, Switzerland: World Health Organization (WHO), 2016. 27977094

[21] National Statistical Office (NSO) [Malawi] and ICF. Malawi Demographic and Health Survey 2015–16. Zomba, Malawi, and Rockville, Maryland, USA. NSO and ICF: Zomba, Malawi, and Rockville, Maryland, USA. NSO and ICF, 2017.

[22] Hayes JR, Moulton LH. Cluster Randomised Trials: Chapman and Hall/CRC; 2009.

[23] Choko AT, Corbett EL, Stallard N, Maheswaran H, Lepine A, Johnson CC, et al. HIV self-testing alone or with additional interventions, including financial incentives, and linkage to care or prevention among male partners of antenatal care clinic attendees in Malawi: An adaptive multi-arm, multi-stage cluster randomised trial. PLoS medicine. 2019;16(1):e1002719. Epub 2019/01/03. doi: 10.1371/journal.pmed.1002719 ; PubMed Central PMCID: PMC6314606 following competing interests: ELC’s institution has received research grants for research into HIV self-testing from Wellcome Trust and Unitaid. RH receives research grants from the UK Medical Research Council, NIH and 3ie. 30601823 PMC6314606

[24] Chipeta MG, Ngwira B, Kazembe LN. Analysis of Schistosomiasis haematobium infection prevalence and intensity in Chikhwawa, Malawi: an application of a two part model. PLoS neglected tropical diseases. 2013;7(3):e2131. Epub 2013/04/05. doi: 10.1371/journal.pntd.0002131 ; PubMed Central PMCID: PMC3605235. 23556017 PMC3605235

[25] R Core team. R: A language and environment for statistical computing. R Foundation for Statistical Computing, Vienna, Austria. URL http://www.R-project.org/. 2015.

[26] Sakala D, Kumwenda MK, Conserve DF, Ebenso B, Choko AT. Socio-cultural and economic barriers, and facilitators influencing men’s involvement in antenatal care including HIV testing: a qualitative study from urban Blantyre, Malawi. BMC public health. 2021;21(1):60. Epub 2021/01/08. doi: 10.1186/s12889-020-10112-w ; PubMed Central PMCID: PMC7789341. 33407298 PMC7789341

[27] Skovdal M, Campbell C, Madanhire C, Mupambireyi Z, Nyamukapa C, Gregson S. Masculinity as a barrier to men’s use of HIV services in Zimbabwe. Global Health. 2011;7(1):13. doi: 10.1186/1744-8603-7-13 ; PubMed Central PMCID: PMC3107786. 21575149 PMC3107786

[28] Siu GE, Wight D, Seeley JA. Masculinity, social context and HIV testing: an ethnographic study of men in Busia district, rural eastern Uganda. BMC Public Health. 2014;14(1):33. doi: 10.1186/1471-2458-14-33 ; PubMed Central PMCID: PMC3893584. 24417763 PMC3893584

[29] Siu GE, Wight D, Seeley J. ’Dented’ and ’resuscitated’ masculinities: the impact of HIV diagnosis and/or enrolment on antiretroviral treatment on masculine identities in rural eastern Uganda. SAHARA J. 2014;11(1):211–21. doi: 10.1080/17290376.2014.986516 ; PubMed Central PMCID: PMC4272191. 25444303 PMC4272191

[30] Izugbara CO, Undie C-C, Mudege NN, Ezeh AC. Male youth and Voluntary Counseling and HIV-Testing: the case of Malawi and Uganda. Sex Education. 2009;9(3):243–59. doi: 10.1080/14681810903059078

[31] Chikovore J, Gillespie N, McGrath N, Orne-Gliemann J, Zuma T, Group ATS. Men, masculinity, and engagement with treatment as prevention in KwaZulu-Natal, South Africa. AIDS care. 2016;28 Suppl 3:74–82. doi: 10.1080/09540121.2016.1178953 ; PubMed Central PMCID: PMC5096677. 27421054 PMC5096677

[32] Chikovore J, Hart G, Kumwenda M, Chipungu GA, Corbett L. ’For a mere cough, men must just chew Conjex, gain strength, and continue working’: the provider construction and tuberculosis care-seeking implications in Blantyre, Malawi. Glob Health Action. 2015;8(1):26292. doi: 10.3402/gha.v8.26292 . 25833138 PMC4382597

[33] Sweeney S, Obure CD, Maier CB, Greener R, Dehne K, Vassall A. Costs and efficiency of integrating HIV/AIDS services with other health services: a systematic review of evidence and experience. Sexually transmitted infections. 2012;88(2):85–99. doi: 10.1136/sextrans-2011-050199 22158934

[34] Hamilton A, Thompson N, Choko AT, Hlongwa M, Jolly P, Korte JE, et al. HIV Self-Testing Uptake and Intervention Strategies Among Men in Sub-Saharan Africa: A Systematic Review. Frontiers in Public Health. 2021;9:60. doi: 10.3389/fpubh.2021.594298 33681120 PMC7933016

[35] Choko AT, Fielding K, Stallard N, Maheswaran H, Lepine A, Desmond N, et al. Investigating interventions to increase uptake of HIV testing and linkage into care or prevention for male partners of pregnant women in antenatal clinics in Blantyre, Malawi: study protocol for a cluster randomised trial. Trials. 2017;18(1):349. doi: 10.1186/s13063-017-2093-2 ; PubMed Central PMCID: PMC5525336. 28738857 PMC5525336

[36] Choko AT, Kumwenda MK, Johnson CC, Sakala DW, Chikalipo MC, Fielding K, et al. Acceptability of woman-delivered HIV self-testing to the male partner, and additional interventions: a qualitative study of antenatal care participants in Malawi. Journal of the International AIDS Society. 2017;20(1):21610. doi: 10.7448/IAS.20.1.21610 ; PubMed Central PMCID: PMC5515040. 28691442 PMC5515040

[37] The CDI Study Group. Community-directed interventions for priority health problems in Africa: results of a multicountry study. Bulletin of the World Health Organization. 2010;88(7):509–18. Epub 2010/07/10. doi: 10.2471/BLT.09.069203 ; PubMed Central PMCID: PMC2897985. 20616970 PMC2897985

[38] Malawi Ministry of Health. Malawi HIV Self-Testing Operational Guidelines. Lilongwe, Malawi: 2018.

[39] MacPherson P, Lalloo DG, Webb EL, Maheswaran H, Choko AT, Makombe SD, et al. Effect of optional home initiation of HIV care following HIV self-testing on antiretroviral therapy initiation among adults in Malawi: a randomized clinical trial. JAMA: the journal of the American Medical Association. 2014;312(4):372–9. doi: 10.1001/jama.2014.6493 ; PubMed Central PMCID: PMC4118051. 25038356 PMC4118051

