# Supplementary material for: Assessing the efficacy of an integrated intervention to create demand for fishermen’s schistosomiasis and HIV services (FISH) in Mangochi, Malawi: Study protocol for a cluster randomized control trial
Source: PLoS One. 2022 Jan 7;17(1):e0262237. doi: 10.1371/journal.pone.0262237 (PMC8741025; doi:10.1371/journal.pone.0262237)
Supplement: S1 File — (DOC) [file pone.0262237.s001.doc]

# Malawi-Liverpool-Wellcome Trust

# Clinical Research Programme

P.O Box 30096, Chichiri, Blantyre 3, Malawi.

Tel. +265 1 876444 Fax +265 1 875774

**Title:** **Creating demand for Fishermen’s schistosomiasis and HIV services (FISH): piloting and delivery of a 3-arm cluster randomized control trial (cRCT) in Malawi**

**Participant ID**

**Principal Investigator:** Dr. Augustine Choko

**Funder:** Wellcome Trust, UK and National Institute for Health Research

**Sponsor:** Liverpool School of Tropical Medicine

**Study site:** Mangochi, Malawi

*[The following text must be read to the participant, who must have their own copy to take home]*

**Introduction**

Hello. My name is ……………………………………………, and I am working with Malawi Liverpool Wellcome Trust (MLW) on behalf of Dr. Augustine Choko and colleagues. We thank you for accepting to answer a few questions about yourself. In this component of the study, we are interested in understanding a few things about fishermen who are part of a cluster randomized trial about increasing demand for HIV and schistosomiasis services.

**Request for your Voluntary Participation**

I would like to ask you to voluntarily participate in this interview. You have been identified because you are a fisherman here in Mangochi. Please note that you were not selected to participate in this interview because you are HIV positive or negative, or that because you have schistosomiasis. We consider that your participation in this project would help us understand the best ways to serve fishing communities with HIV and schistosomiasis services. This is very important because the Ministry of Health may consider implementing successful strategies studied here nationally.

**Procedure / what the study involves**

Please note that we are carrying out these procedures on everyone who accepts to participate in this interview. All participants are asked a few questions about themselves and/or their social and sexual networks.

Your participation is entirely voluntary. If you decide to take part, you may withdraw from the interview any time. You also have a right not to answer any particular question or questions that will be asked. Declining to participate in this interview will not affect any health services that you or any person related to you may be currently receiving or may require in future. You have a day to decide if you want to take part or not in this study.

You will be offered a coupon during study participation depending on the arm in which your landing will be randomized to. Should you be in the standard of care arm, your peer leader will offer you a coupon which you can use to access HIV and schistosomiasis services at the beach clinic which will be set up very close to your landing site. He will also drop a leaflet explaining HIV and schistosomiasis issues on your boat. If you are in the peer educator arm, your peer leader will offer you a coupon and explain the leaflet covering HIV and schistosomiasis issues. If you are in the peer distributor educator arm, the peer leader will offer you a coupon, an HIV self-test kit and explain the leaflet covering HIV and schistosomiasis issues. Please note that you have a right to refuse any of these services and that doing so will not attract any punishment from the peer leader, researchers or health workers now or in the future. If you feel coerced by the peer leader to use any of these services, then kindly report this to the study team or the ethics committee as detailed below.

The baseline interview will take no more than 10 minutes. Then the main part of the study will take one month with a final interview lasting about 20 minutes nine months after the baseline interview. The consent you will give now will be for the entire duration of the study if you decide to participate. Should you decide to withdraw from any part of the study, you will still be able to access the beach clinic services but as a general community member. Should this premature withdraw happen, we will only use part of the data for which consent was given, such as the baseline data.

**Confidentiality**

All personal information collected in this study will be kept strictly confidential. I will not share the information you provide with anyone who is not part of this research. But it may be shared with fellow researchers and may also be published through meetings or journals in a manner that does not reveal your identity. Before sharing in this manner, the information from you will be combined with that from other research participants. Information which could identify you or anyone related to you will never be released. This also means that names of study participants, including your own will not be included when sharing the data. Data collection equipment and the data collected will be kept with identifiers, locked, and only accessible to people that have authorised access.

**Risks**

You may be uncomfortable with some of the questions that I will ask. You are perfectly entitled to refuse to discuss issues that you do not want to.

If you accept to take treatment for schistosomiasis, you may experience some side effects. Common side effects for praziquantel are headache, nausea, abdominal pain, dizziness, drowsiness, fatigue, weakness, joint/muscle pains, loss of appetite, vomiting, sweating, itching. These are usually mild and transient, lasting from 30 minutes to up to 4 hours. You need to take adequate fluids, rest and take praziquantel after evening meal as you go to bed. Serious side effects which are very rare include bloody diarrhoea, fever, irregular or slow heart beat or seizures/convulsions.

There are no side effects to using oral HIV self-test kits. However, if you experience a misunderstanding between you and the peer distributor educator leading to physical violence or abuse, kindly report it to the researchers through the contact number provided below.

**Benefits**

There are no direct benefits to you in your taking part in this interview. However, what we learn from this study would help the Ministry of Health to make important decisions regarding how best to serve fishing communities with HIV and schistosomiasis services. It is not yet known if services including the offer of HIV self-test kits would increase demand for HIV and schistosomiasis services. Therefore, your participation would benefit many others in the future.

**Compensation**

You will not receive payment for participating in the study.

**Contact details**

This research has been approved by the College of Medicine Research Ethics Committee (COMREC) and the Liverpool School of Tropical Medicine Research Ethics Committee. If you have any questions regarding your rights as a research participant, or concerns on how you have been treated in the study, please feel free to contact **Dr.** **Augustine Choko** [+265 (0) 999 577 452] or [augutc@gmail.com]. If you have any questions regarding your rights as a research participant, or concerns on how you have been treated in the study, please feel free to contact COMREC Secretariat, College of Medicine, Private Bag 360, Chichiri, Blantyre 3 or call on 01871911 ext 334.

**Consent Declaration**

If you agree to voluntarily participate in the study, please sign or write your initial or your thumb print below to show that you understand the information above and that your consent is given voluntarily.

|  |  | Participant  Initials / thumb print |
| --- | --- | --- |
| 1 | I have received and read or had read to me the information sheet C02a: Participant Information and Consent Form for Baseline Interview - Eng v1.3 of 26 Aug 2020 provided by the Researcher that explains in detail the reasons for the study. | --------------------------- |
| 2 | I have understood the purpose of the research. | --------------------------- |
| 3 | I have asked all the questions that I have about the purpose of the research and feel that I have enough information about it. | --------------------------- |
| 4 | I understand the reasons for this study. | --------------------------- |
| 5 | I am willing to take part in the study. | --------------------------- |
| 6 | I understand what I will be required to do if I participate in the study. | --------------------------- |
| 7 | I know that I have the right to leave the study at any time or to refuse to answer any questions. | --------------------------- |
| 8 | If I do not agree to take part in this study, I understand that I will not be penalized for doing so by the researcher nor by any medical service providers in the future. | --------------------------- |
| **9** | **I voluntarily agree to take part in this study** | --------------------------- |

------------------------------------------------- -----/-------/-------- -----------------------------------

Participant name Date Signature or thumb print

**If the participant gave verbal consent, please enter the name of person who witnessed the consent here, and their signature:**

------------------------------------------------- -----/-------/-------- -----------------------------------

Name of Witness (BLOCK CAPITALS) Date Signature or thumb print

------------------------------------------------- -----/-------/-------- -----------------------------------

Name of person obtaining consent Date Signature
