# Supplementary material for: Assessing the efficacy of an integrated intervention to create demand for fishermen’s schistosomiasis and HIV services (FISH) in Mangochi, Malawi: Study protocol for a cluster randomized control trial
Source: PLoS One. 2022 Jan 7;17(1):e0262237. doi: 10.1371/journal.pone.0262237 (PMC8741025; doi:10.1371/journal.pone.0262237)
Supplement: S2 File — (DOCX) [file pone.0262237.s002.docx]

**Creating demand for Fishermen’s schistosomiasis and HIV services (FISH): piloting and delivery of a 3-arm cluster randomized control trial (cRCT) in Malawi (FISH)**

**Statistical Analysis Plan**

(Linked to Protocol v1.4; 11 Jan 2021)

**Principal investigator:** Dr Augustine Talumba Choko (MLW)

**Funder:**

**Co-investigators:**

| Prof Katherine Fielding | London School of Hygiene & Tropical Medicine (LSHTM), London, UK |
| --- | --- |
| Prof Elizabeth L Corbett | LSHTM & Malawi Liverpool Wellcome Trust Clinical Research Programme (MLW), Blantyre, Malawi |
| Prof Antony Butterworth | MLW |
| Dr Peter MacPherson | Liverpool School of Tropical Medicine (LSTM), Liverpool, UK & MLW |
| Dr Moses Kumwenda | MLW & Kamuzu University of Health Sciences, Blantyre, Malawi |
| Dr Sekeleghe Kayuni | LSTM & Medical Aid Society of Malawi (MASM) |
| Prof Russel Stothard | LSTM |
| Prof Stephane Helleringer | New York University, Abu Dhabi |
| Ms Cheryl Johnson | HIV Department, WHO, Geneva, Switzerland |
| Dr Hendramoorthy Maheswaran | ‎Imperial College London, London, UK |
| Mrs Rose Nyirenda | Department of HIV/AIDS, Lilongwe, Malawi |
| Mr Lazarus Juziwelo | schistosomiasis Control Programme, Lilongwe, Malawi |

**Institutions:**

| London School of Hygiene and Tropical Medicine (LSHTM), London, UK |
| --- |
| Malawi Liverpool Wellcome Trust Clinical Research Programme (MLW), Blantyre, Malawi |
| Liverpool School of Tropical Medicine, Liverpool, United Kingdom |
| schistosomiasis Control Programme, Lilongwe, Malawi |
| Ministry of Health, Department of HIV/AIDS, Lilongwe, Malawi |
| New York University, Abu Dhabi |
| Kamuzu University of Health Sciences |
| HIV Department, World Health Organization, Geneva, Switzerland |
| Imperial College London, London, UK |

1. **Overview of study design, setting and recruitment.**

This is a 3 – arm cluster randomised trial aimed to investigate whether secondary distribution of HIV self-tests through peers (selected fishermen) can increase demand for HIV services and schistosomiasis presumptive treatment offered via a beach clinic on the lakeshore among fishermen. It has three arms, 1) Standard of care (SOC arm) with written invitation and a study leaflet to the boat crew 2) SOC plus active explanation and encouragement to attend the beach clinic by a peer educator – a selected boat crew member (PE arm) 3) PE plus distribution of oral HIV self-test kits by a peer distributor educator who is a member of the boat crew (PDE arm).

1. **Outcomes**

**Primary outcomes**

Primary outcomes measured at 9 months of trial delivery will compare differences between arms in the **proportions of boat-team fishermen**: -

1. Who self-report starting ART or undergoing VMMC during.
2. Who have ≥1 *S. haematobium* egg seen on light microscopy of the filtrate from 10mls urine (“egg-positive”).

**Secondary outcomes**

Secondary outcomes will compare differences between arms in: -

1. Self-reported recent (last 9 months) HIV testing
2. Self-reported HIV prevention knowledge score
3. Self-reported schistosomiasis knowledge score
4. Self-reported high-risk sex in the last month
5. *S. haematobium* intensity
6. **Randomisation**

The unit of randomization is boat teams where a boat team is defined as a landing site or part of land from which fishermen dock. Boat teams were randomised 1:1:1 using computerized restricted block randomization (geographical spread, cluster size, traditional authority, and HIV and schistosomiasis estimates) at a public randomisation ceremony. One allocation was selected at random from 39,262 possible allocations following restriction with final arm assignment completed at the public ceremony held on Friday 28 May 2021. Cluster representatives attended the public randomization ceremony and participated in the arm assignment exercise.

1. **Description of each trial arm**
2. Standard of care (SOC) arm: selected boat crew members from SOC clusters will be given invitation letters and leaflets to give to their boat crew members. Any boat crew member would then present this pre-allocated invitation letter at the beach clinic in order to receive available HIV services and presumptive schistosomiasis treatment.
3. Peer-educator (PE) arm: In addition to invitation letters and information leaflets provided in the SOC arm, peer educators will actively explain the leaflet and encourage fellow boat crew members in clusters from this arm to attend the beach clinic.
4. Peer-distributor-educator (PDE) arm: Selected boat crew members called peer distributor educators will follow PE arm procedures with the addition of providing oral HIVST kits to their fellow boat crews. PDEs will be trained in HIVST for secondary distribution. Information and educational materials demonstrating correct use of kits will be part of the training.
5. **Sample size justification**

Using established cluster-randomised methodology(1) 15 boat-teams/arm (1,500 fishermen/arm) would provide 80% power to detect a 9% increase in combined ART/VMMC uptake compared to an assumed 10% under SOC(2) for the first primary outcome. We assumed intercluster coefficient of variation (*k*) of 0.20, with HIVST uptake of 50%-80%(2, 3) (Table 1).

For the **second primary** outcome, we assumed praziquantel uptake will be 10% to 20% higher in PDE/PE arms than an assumed 40-60% for the SOC arm**,** with baseline egg-positivity 15-25%, 95% cure from praziquantel, and *k* between 0.20-0.30.(4, 5) Power provided by the 15 clusters/arm for HIV endpoints was >80% over most of this range of scenarios.

**Table 1:** Assumptions and parameters for sample size

| **Assumptions** | |
| --- | --- |
| Average cluster size (number of men in a fishing dock) | 100 |
| Proportion eligible for the trial (mainly not already on ART) | 0.80 |
| Proportion accepting to self-test | 0.50 – 0.80(2, 3) |
| Proportion HIV positive or HIV negative and uncircumcised | 0.20 – 0.50(2) |
| Uptake of praziquantel in SOC arm by endline | 0.4-0.6 |
| Proportion urine egg-positive in SOC arm (endline) | 0.09-0.17 |
| Geometric mean egg-count if egg-positive in SOC (endline) | 10/10mL |
| **Parameters** | |
| Significance level (α) | 0.05 |
| Power (1-β) | 0.80 |
| Allocation ratio | 1:1:1 |

1. **Trial profile**

A trial profile figure based on the extension of the CONSORT for cluster randomised trials will be produced illustrating the following (Figure 1):

1. Overall number of clusters and number of fishermen assessed for eligibility.
2. Number of clusters excluded; number of fishermen excluded because of being ineligible with reasons.
3. Overall number of clusters and number of fishermen randomly assigned.
4. Number of clusters allocated to each arm.
   1. Number of fishermen who met inclusion criteria in each arm.
   2. Number of fishermen who were recruited in each arm.
   3. Number of fishermen who did not consent to participate in each arm.
5. Number of clusters and fishermen lost to follow up in each arm.
6. Number of fishermen who completed follow up in each arm.
7. Number of clusters and fishermen included in primary analysis each arm.
8. **Baseline enrolment characteristics**

Analysis will be done in R and Stata 14.0 (Stata Corp, Texas, USA). Baseline characteristics will be computed as proportions or median (interquartile range [IQR]), as appropriate, by arm (Table 1).

Some individual level characteristics will include:

Age

Ability to read

Education

HIV testing history

Previous use of schistosomiasis treatment

1. **Statistical analysis of primary outcome**

A test of null hypothesis of no difference in effectiveness of each intervention compared to SOC will be conducted. All analyses will be done by intention to treat. The clustered design will be taken into account during analysis. In each cluster the proportion of fishermen achieving the primary outcome (s) will be calculated (Table 2).

$$\frac{number of fishermen achieving the primary outcome (s)}{number of fishemen eligible}$$

Cluster level summaries will be examined graphically in each trial arm to determine the distribution of the per cluster proportion with the primary outcome (s). If the distributions are skewed, logarithm will be applied to approximate normal distributions. The mean of proportions in each of the two intervention arms will be compared to the SOC arm using unpaired t-test. A risk ratio (RR) and 95% CI will be computed for each comparison by dividing the mean of proportions in each intervention arm and the mean of proportions in the SOC arm. A random effects logistic regression model will also be fitted to the individual level data to estimate the odds ratio (OR) and 95% CI for each of the intervention arms. These models will also be used to estimate the coefficient of variation (*k*) for each of the two primary outcomes.

1. **Analysis of secondary outcomes.**

The analysis all secondary outcomes that are measured as a proportion will proceed in a similar fashion to the analysis of the primary outcomes as described above (Table 3). For the HIV and schistosomiasis knowledge outcomes, a binary outcome coded as 1=for those showing knowledge and 0=for those without adequate knowledge will be first generated based on the median knowledge score.

The secondary outcome of *S. haematobium* intensity will begin with a histogram plotted to examine the distribution of eggs per 10ml of urine. The geometric mean and standard deviation of eggs per 10ml urine will then be computed for each trial arm. An incidence rate ratio (IRR) and 95% CI will be computed by fitting a negative binomial model.

1. **Sources of bias and contamination**

Potential sources of bias which will be examined including:

- Contamination between clusters: it is possible for fishermen from the SOC arm to benefit from the interventions such as by getting self-test kits or direct and indirect encouragement to attend the beach clinic. We will examine the proportion of participants in the SOC arm who report having received a self-test kits.

Figure 1: Trial Profile

Assessed for eligibility (n = 45 clusters); N = xxxx fishermen

Total (n=)

Total number excluded (n =)

Not eligible after eligibility screening (n=)

No consent given but eligible (n =)

**Standard of care arm**

Total (n=)

Randomisation (n = 45 clusters)

Lost to follow-up

(n = 0 clusters)

Total (n=)

Enrolment

**PE arm**

Total (n=)

**PDE arm**

Total (n=)

Lost to follow-up

(n = 0 clusters)

Lost to follow-up

(n = 0 clusters)

Total (n=)

Allocation

Follow-up

Analysis

**Table 1:** Baseline characteristics of fishermen by trial arm (N = XXXX)

|  | **Trial arm** | | | |
| --- | --- | --- | --- | --- |
| **Variable** | **Characteristic** | **SOC** | **PE** | **PDE** |
| Number responded | n |  |  |  |
| Age (years) | Mean (sd) |  |  |  |
| Age group | 15 – 19  (20,30]  (30,60] |  |  |  |
| Able to read | Yes  No |  |  |  |
| HIV testing history | Never tested before |  |  |  |
|  | Tested before |  |  |  |
| Previous use of praziquantel | Yes |  |  |  |
|  | No |  |  |  |

SOC: standard of care; PE: peer educator; PDE: peer distributor educator; sd: standard deviation

**Table 2:** Intervention effects by trial arm (**primary outcomes**)

|  | **Trial arm** | | |
| --- | --- | --- | --- |
|  | **SOC** | **PE** | **PDE** |
| Eligible |  |  |  |
| Outcome* |  |  |  |
| Proportion** |  |  |  |
| RR |  |  |  |
| 95% CI** |  |  |  |
| P – value |  |  |  |
| Outcome |  |  |  |
| Mean |  |  |  |
| RR |  |  |  |
| 95% CI |  |  |  |
| P – value |  |  |  |

SOC: standard of care; PE: peer educator; PDE: peer distributor educator; RR: risk ratio

*Fishermen achieving the primary outcome (s)

** mean of the cluster proportions

**Table 3:** Intervention effects by trial arm **(secondary outcomes)**

|  | **Trial arm** | | |
| --- | --- | --- | --- |
|  | **SOC** | **PE** | **PDE** |
| Eligible |  |  |  |
| Secondary outcome* |  |  |  |
| Proportion |  |  |  |
| RR |  |  |  |
| 95% CI |  |  |  |
| P – value |  |  |  |
| Secondary outcome† |  |  |  |
| Mean |  |  |  |
| IRR |  |  |  |
| 95% CI |  |  |  |
| P – value |  |  |  |

SOC: standard of care; PE: peer educator; PDE: peer distributor educator; RR: risk ratio; IRR: incidence rate ratio

* Fishermen achieving the primary outcome (s)

† Geometric mean number per cluster

**References**

1. Hayes JR, Moulton LH. Cluster Randomised Trials: Chapman and Hall/CRC; 2009.

2. Choko AT, Corbett EL, Stallard N, Maheswaran H, Lepine A, Johnson CC, et al. HIV self-testing alone or with additional interventions, including financial incentives, and linkage to care or prevention among male partners of antenatal care clinic attendees in Malawi: An adaptive multi-arm, multi-stage cluster randomised trial. PLoS medicine. 2019;16(1):e1002719.

3. Choko AT, Nanfuka M, Birungi J, Taasi G, Kisembo P, Helleringer S. A pilot trial of the peer-based distribution of HIV self-test kits among fishermen in Bulisa, Uganda. PLoS One. 2018;13(11):e0208191.

4. Kayuni S, Peeling R, Makaula P. Prevalence and distribution of Schistosoma haematobium infection among school children living in southwestern shores of Lake Malawi. Malawi medical journal : the journal of Medical Association of Malawi. 2017;29(1):16-23.

5. Chipeta MG, Ngwira B, Kazembe LN. Analysis of Schistosomiasis haematobium infection prevalence and intensity in Chikhwawa, Malawi: an application of a two part model. PLoS neglected tropical diseases. 2013;7(3):e2131.
