# Supplementary material for: Assessing the efficacy of an integrated intervention to create demand for fishermen’s schistosomiasis and HIV services (FISH) in Mangochi, Malawi: Study protocol for a cluster randomized control trial
Source: PLoS One. 2022 Jan 7;17(1):e0262237. doi: 10.1371/journal.pone.0262237 (PMC8741025; doi:10.1371/journal.pone.0262237)
Supplement: S3 File — (DOCX) [file pone.0262237.s003.docx]

**Creating demand for Fishermen’s schistosomiasis and HIV services (FISH): piloting and delivery of a 3-arm cluster randomized control trial (cRCT) in Malawi**

**Malawi Investigators**

Dr Augustine Talumba Choko^1^: Principal Investigator

Prof Elizabeth Corbett^1, 2^; Prof Antony Butterworth^1^: Epidemiological and trial design input

Dr Moses Kumwenda^1^: Social Science input, Dr Peter MacPherson^1, 3^: General trial input

Dr Sekeleghe Kayuni^3, 4^: Clinical input for schistosomiasis

**Ministry of Health (MoH), Malawi**

Dr Rose Nyirenda^6^: Director of HIV Department

Mr Lazarus Juziwelo^7^, Acting Director of the schistosomiasis Control Programme

**International Collaborators:**

Prof Katherine Fielding^2^: Statistical and epidemiological input

Prof Russel Stothard^3^: Schistosomiasis diagnostics input

Dr Hendramoorthy Maheswaran^3^: Health economics.

Associate Prof Stephane Helleringer^5^ and Dr Guy Harling^6^: Social networks input

Ms Cheryl Johnson^7^, Technical Officer, HIV Department, WHO

**Collaborating Institutions:**

1. Malawi Liverpool Wellcome Trust Clinical Research Programme (MLW), Blantyre, Malawi
2. London School of Hygiene and Tropical Medicine (LSHTM), London, United Kingdom
3. Liverpool School of Tropical Medicine, Liverpool, United Kingdom
4. Medical Aid Society of Malawi (MASM), Blantyre, Malawi
5. John Hopkins University, Baltimore, USA
6. University College London, London, UK
7. HIV Department, World Health Organization, Geneva, Switzerland

**Funder:** Wellcome Trust, UK (International Intermediate Fellowship)

**Sponsor:** Liverpool School of Tropical Medicine is the main research sponsor for this study. For further information regarding the sponsorship conditions, please contact the Research Governance and Ethics Office:

Liverpool School of Tropical Medicine; Pembroke Place; Liverpool; L3 5QA

E-mail: [lstmgov@lstmed.ac.uk](mailto:lstmgov@lstmed.ac.uk); Tel:  00 44 151 705 3796 Fax:  00 44 151 705 3370

This trial will adhere to the principles outlined in the International Council for Harmonisation Good Clinical Practice (ICH GCP) guidelines, protocol and all applicable local regulations.

**Version 1.4 last amended 11 Jan 2021 by ATC**

Signature: **
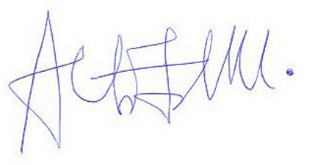
** Date: 11 Jan**2021**

Table of Contents

[**1.** **Executive summary** 4](#_Toc33508912)

[**2.** **Background** 6](#_Toc33508913)

[**2.1.** **Introduction** 6](#_Toc33508914)

[**2.2.** **Background studies leading to the project** 7](#_Toc33508915)

[**3.** **Study rationale** 9](#_Toc33508916)

[**4.** **Research questions, aim and objectives** 9](#_Toc33508917)

[4.1 Research questions 9](#_Toc33508918)

[4.2 Aim and objectives 10](#_Toc33508919)

[**5.** **Methods** 10](#_Toc33508920)

[5.1. Study design and setting 10](#_Toc33508921)

[5.2. Study population 12](#_Toc33508922)

[5.3. Baseline and endline surveys 12](#_Toc33508923)

[5.3.1. Mapping, baseline survey and enumeration 12](#_Toc33508924)

[5.3.2. Endline survey and data extraction 13](#_Toc33508925)

[5.4. Randomisation and blinding 13](#_Toc33508926)

[5.4.1. Randomisation 13](#_Toc33508927)

[5.4.2. Blinding 14](#_Toc33508928)

[5.5. Primary outcomes and measurement 14](#_Toc33508929)

[5.6. Secondary outcomes and measurement 14](#_Toc33508930)

[5.7. Trial procedures 14](#_Toc33508931)

[5.7.1. Trial implementation 14](#_Toc33508932)

[5.7.2. Recruitment and training of peer-distributors/educators (PDEs and PEs) 15](#_Toc33508933)

[5.7.3. Serious adverse events 15](#_Toc33508934)

[5.8. Beach clinics and laboratory methods 16](#_Toc33508935)

[5.9. Sample size considerations 16](#_Toc33508936)

[5.10. Risk mitigation and contingency 17](#_Toc33508937)

[**6.** **Sub-studies** 17](#_Toc33508938)

[6.1. Economic evaluation 17](#_Toc33508939)

[6.2. Social science 18](#_Toc33508940)

[6.3. Social networks 19](#_Toc33508941)

[**7.** **Timelines** 20](#_Toc33508942)

[**8.** **Data management and statistical analysis** 21](#_Toc33508943)

[8.1. Data management 21](#_Toc33508944)

[8.2. Statistical analysis 21](#_Toc33508945)

[**9.** **Piloting** 22](#_Toc33508946)

[**10.** **Ethics, conflict of interest, data availability and dissemination** 22](#_Toc33508947)

[10.1. Ethical approval 22](#_Toc33508948)

[10.2. Sponsor 23](#_Toc33508949)

[10.3. Conflict of interest 23](#_Toc33508950)

[10.4. Compensation for participants 23](#_Toc33508951)

[10.5. Data availability 24](#_Toc33508952)

[10.6. Dissemination 24](#_Toc33508953)

[**11.** **Training and capacity building** 24](#_Toc33508954)

[**12.** **Personnel, materials and consumables, equipment, space** 24](#_Toc33508955)

[12.1. Personnel 24](#_Toc33508956)

[12.2. Materials and consumables 25](#_Toc33508957)

[12.3. Equipment 25](#_Toc33508958)

[12.4. Space 25](#_Toc33508959)

[12.5. Miscellaneous 25](#_Toc33508960)

[**13.** **Budgetary estimate** 26](#_Toc33508961)

[**14.** **References** 27](#_Toc33508962)

[**15.** **Appendices** 30](#_Toc33508963)

[15.1. Tools for FISH project 30](#_Toc33508964)

[15.2. Potential landing sites (clusters) in Mangochi 31](#_Toc33508965)

## **Executive summary**

**Type of study:** This proposal is for a 3-arm project including formative work and a cluster randomised trial (CRT) using a “boat team” as the unit of randomisation.

The three arms are: 1) Standard of care (SOC) with leaflets explaining the importance of receiving presumptive treatment for schistosomiasis (praziquantel) and HIV services for fishermen, and two intervention arms of 2) SOC + a peer explaining the leaflet to his fellow fishermen in a boat team; and 3) arm 2 with HIV self-test kits delivered to the boat team fishermen by the peer.

**Problem:** Both HIV and schistosomiasis are major public health problems worldwide with 1.8 million new HIV infections, and up to 110 million untreated schistosomiasis cases globally. The Rift Valley in Africa suffers high prevalence of both HIV and schistosomiasis, and although the pathology is different, HIV and *Schistosoma* haematobium have important commonalities. First, both are hazards for fishermen with diagnosis being a major barrier to treatment. Secondly, recent studies have suggested that female genital schistosomiasis increases the risk of HIV infection 3 to 4 times in women and although the link between HIV and genital schistosomiasis is not yet established in men there is biological plausibility. Thirdly, controlling HIV in fishing communities may also require tackling schistosomiasis because while policies are in place their implementation is suboptimal.

**Role of MoH, implementers and researchers:** The HIV/AIDS Department of MoH will support implementation of HIVST distribution as it is their aim to scale-up secondary distribution of HIVST. The schistosomiasis Control programme will provide the praziquantel for use during the project. The integrated nature of service provision targeting a hard to reach population is the biggest attraction for both the HIV and schistosomiasis programmes.

**Research Question:** Can peer-educators, with or without HIV self-test kits, increase uptake of services for schistosomiasis and HIV by adult (aged >18 years) fishermen?

**Objectives:** The aim of this project is to identify optimal models of delivering integrated HIV and schistosomiasis services for fishermen, particularly investigating the effect of using social networks, HIV self-test kits and beach clinic services.

The specific objectives for the project are:

1. To conduct formative research to establish baseline data, identify peer- and sexual- networks in the intended study setting, optimise training and educational materials, and optimise HIV and praziquantel service delivery models.
2. To conduct a 3-arm cluster randomized trial (CRT) with a “boat-team” as the unit of randomization, comparing health and knowledge outcomes when outreach Beach Clinic services are promoted by peer educators (PE), or peer-HIVST distributor-educators (PDE), or standard beachside approaches. The primary outcomes will be
   1. uptake of either VMMC or ART during the intervention, and
   2. active *S. haematobium* infection (urine-positive for schistosome eggs).
3. To undertake within-trial economic evaluation to estimate costs and cost-effectiveness.

**Methodology**

*Participants and intervention*

Boat team members (fishermen) resident in 45 landing sites in Mangochi district will be targeted. Restricted randomisation will be used to allocate boat teams to the 3 arms (1:1:1). Peer educators (fishermen selected through a formative phase) will be trained in use of oral HIVST kits (OraQuick). Information and educational materials demonstrating correct use of kits will be part of the training. A beach clinic, providing HIV testing, confirmatory testing and referral for ART and voluntary male medical circumcision will be set up. The beach clinic will also provide presumptive praziquantel to all attendees. Demand for beach clinic services will be through mere provision of leaflets in the SOC arm, active explanation and encouragement from a peer fisherman educator with or without provision of the HIVST kits. Data capture tools and training materials will be developed with MoH.

Full-time HIV counsellors and lab techs will man the beach clinic whereas a nurse seconded from Mangochi district hospital will manage the administration of praziquantel with clinical oversight by a Doctor (co-investigator).

*Primary and secondary outcomes:*

Primary outcomes measured at 9 months of trail delivery will compare differences between arms in the **proportions of boat-team fishermen**: -

1. Who self-report starting ART or undergoing VMMC during.
2. Who have ≥1 *S. haematobium* egg seen on light microscopy of the filtrate from 10mls urine (“egg-positive”).

Secondary outcomes will compare differences between arms in: -

1. Self-reported recent (last 9 months) HIV testing
2. Self-reported HIV prevention knowledge score
3. Self-reported schistosomiasis knowledge score
4. Self-reported high risk sex in the last month
5. *S. haematobium* intensity

*Sample size:*

Using established cluster-randomised methodology 15 boat-teams/arm (1,500 fishermen/arm) will provide 80% power to detect a 9% increase in combined ART/VMMC uptake compared to an assumed 10% under SOC. We assume intercluster coefficient of variation (*k*) of 0.20, with HIVST uptake of 50%-80%.

**Ethical considerations:** We request a waiver of informed consent for HIVST, as this is now national policy and international best practice. Written informed consent (witnessed thumb-print if illiterate) will be taken from all participants in the qualitative study, and the endline survey.

**Dissemination:** The results will be used to inform MoH on HIVST scale-up plans and integration of HIV and schistosomiasis services for fishermen, and will also be disseminated through College of Medicine in Blantyre including COMREC, and through conference presentations and publication in peer-reviewed journal.

## **Background**

## **Introduction**

HIV prevalence in Malawi is among the highest globally, with 9.6% of the adult population living with HIV, and 39,000 new infections and 17,000 deaths from HIV in 2017^1^. Remarkable progress has been made towards meeting the 2020 UNAIDS “90-90-90” targets for the HIV care cascade: population-based surveys in 2016 estimated that 72.7% of Malawian people living with HIV (PLHIV) were diagnosed, of whom 88.6% had started antiretroviral treatment (ART), with 90.8% of those virally suppressed^2^. As well as substantially individual health benefits, viral suppression profoundly reduces infectiousness (“treatment-as-prevention”)^3-6^. Increased ART coverage has reduced annual new infections and deaths in Malawi by an estimated 39% and 50% since 2017^3^, despite little investment in other HIV prevention modalities. Awareness of effective HIV prevention approaches remains low; even in young people, 72.2% of men remain uncircumcised, and condom use is suboptimal even with non-primary/transactional partners^1^.

Fishing communities throughout Africa and Asia have much higher HIV prevalence than national averages with, for instance, complex ‘fish-for-sex’ trading networks described in Malawi^4^. From the male perspective, fishing jobs with the highest risk of drowning typically pay a share of the catch to low-status migrant labourers, for whom risk-taking behaviour with alcohol and transactional sex can then provide temporary relief from the dangerous, physical demands and insecurity of their work^4^. For women, transactional sex can bring favourable access to quality fish and a way to recover lost capital from unsold spoilt fish^4^.

In freshwaters, including Lake Malawi, urinary schistosomiasis (*Schistosoma haematobium*) is a further occupational hazard and, among other pathologies, commonly causes male and female genital lesions resulting in subfertility, haemospermia, genital and pelvic pain^7,8^. Although a causal link has not been definitely established, there are strong suggestions that having schistosomiasis increases onward transmission of HIV from co-infected men to women, and also that schistosomiasis increases susceptibility to HIV in HIV-negative women.^7,8^ Most recently, stored serum showed 59% baseline schistosome-antibody positivity in a large Zambian cohort of HIV discordant couples (i.e. only one partner HIV-positive at baseline) that documented 335 HIV seroconversion events during follow-up^8^. Baseline HIV-positive schistosome-positive partners were more likely to transmit HIV to their HIV-negative partner (adjusted hazard ratios 1.8 for men and 1.4 for women), with HIV-negative women who had serological evidence of *S. haematobium* at increased risk of acquiring HIV (adjusted hazard ratio 1.4)^8^.

International recommendations for high prevalence communities include annual treatment with praziquantel for at-risk adults, such as fishermen, as well as annual mass drug administration (MDA) for school age children^9^. Malawi has delivered several MDA campaigns but mainly to children^10^. A 2018 survey showed poor control in lakeshore men, with 17% having *S. haematobium* eggs in urine (personal communication with Dr Seke Kayuni). Engaging adults in MDA programmes will be essential for elimination^11^, since schistosomes are long-lived helminth parasites capable of producing eggs for decades if untreated^7^. Egg output depends on the number of mating parasite pairs within the host. Eggs migrate through tissues and are either retained, provoking granuloma formation, or excreted in urine, leading to infection of fresh-water snails. Once treated, acquired immunity results in lasting cure or greatly reduced intensity of reinfection of almost all adults, even if HIV-positive and with ongoing water contact^12^ although symptoms may continue due to retained eggs and fibrosis^7^.

With both HIV and schistosomiasis treatment readily available in Malawi, we propose to investigate the feasibility, acceptability and health impacts of joint management of these two hazards, with special focus on health education and demand-creation for fishermen. As elsewhere, Malawian men have delayed health-seeking relative to women, with lower coverage of lifetime HIV testing (69.9% vs 83.3% in women^2^ and more advanced HIV at ART initiation^13^.

Harmful masculine norms relating to health service usage ^14-17^ in part explain this, but men also face substantial access barriers especially when trying to access clinic services from a position of job insecurity^18,19^. Clinics tend to be structured around maternal and child health in a way that unintentionally alienates and disadvantages men^18,19^.

## **Background studies leading to the project**

Malawi Liverpool Wellcome Trust Clinical Research Programme has led the rapid global scale-up of HIV self-testing (HIVST). Our previous Wellcome-funded work on partner-delivered HIVST showed HIVST to be strongly preferred over other testing modalities, with high uptake (87.0% to 95.4%) of men self-testing using kits delivered by their pregnant partner (Figure 1a)^20-22^. Incentives did not affect HIVST uptake but did substantially increase uptake of VMMC and ART (Figure 1b)^20^. Our recent pilot study in Uganda showed high acceptability (82% of 116 men offered a kit accepting to self-test) and safety of peer-delivered HIVST to fishermen^23^.

Community-led MDA and peer-based interventions can reach remote or hard-to-reach populations more effectively than standard interventions^24^. Malawi National Policy already supports praziquantel presumptive treatment and peer-based HIVST delivery for sex-workers and male workplaces^25^. Delivering HIVST along with additional interventions can encourage prompt uptake of VMMC and ART^20,26^.

Novel aspects are that this will be the first trial to directly investigate whether the high enthusiasm and engagement shown by men towards HIVST kits can be used to increase demand for a less desirable intervention (schistosomiasis), increasing cost-effectiveness. It is also possible that HIVST will instead distract from praziquantel. This will also be one of the few randomised trials in any field using advanced network theory, investigating broader benefits to social networks, and directly comparing intervention arms with and without engagement of social networks^27-29^.

**Figure 1a and 1b:** Uptake of male partner HIV-testing and ART/VMMC within 28 days (2,349 women): Choko PLos Med 2019^20^


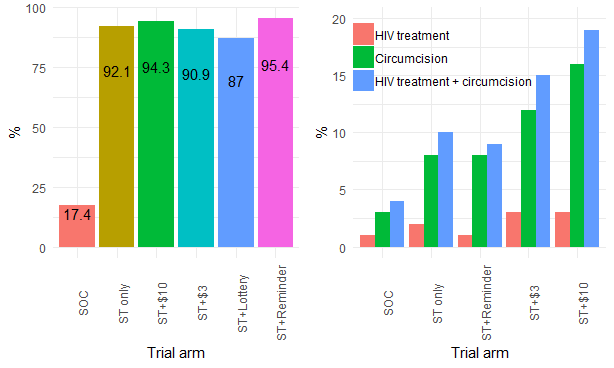


SOC: standard of care; ST: self-test kits

Lottery: self-test kits and 10% chance of winning US$30 upon achieving the primary outcome

Reminder: self-test kits and phone call to the male partner

For Fig. 1b: clinic attendance ranged from 19% to 52% for the HIV self-testing arms.

## **Study rationale**

Prevalence of HIV remains unusually high in fishing communities yet services miss the intended targeted population of fishermen due to the nature of their occupation. Similarly, MDA with praziquantel targeting fishermen is not optimally delivered for lack of availability of the fishermen. This project builds on a previous pilot trial in Uganda which explored secondary distribution of HIV self-test kits via peers of fishermen^23^. Secondary distribution of HIVST kits is a WHO recommended approach for increasing testing particularly for men who are hard to reach such as fishermen^30^. It is also believed that having schistosomiasis may increase the risk of HIV transmission and acquisition in men. Such a risk, although not yet established albeit with biological plausibility^31^, implies that there is urgent need to find optimal models of increasing demand for HIV and schistosomiasis services among fishermen. Thus, this project aims to test peer-based approaches of increasing demand for HIV and schistosomiasis services among fishermen in Mangochi with or without the offer of HIVST kits.

## **Research questions, aim and objectives**

### Research questions

Can peer-educators, with or without HIV self-test kits, increase uptake of services for schistosomiasis and HIV by adult (>18 years) fishermen?

Do peer-based interventions improve biological (*S. haematobium* eggs/10 mls urine) or self-reported (VMMC if HIV-negative, ART if HIV-positive) health states?

Do peer-based interventions improve understanding of HIV prevention and the benefits of early treatment of HIV and *S. haematobium*?

Do peer-based interventions bring benefits (recent HIV testing, praziquantel, knowledge scores) to social networks? And are there unintended consequences, such as intimate partner violence or reduced uptake of praziquantel, when HIVST is provided?

### Aim and objectives

The aim of this project is to identify optimal models of delivering integrated HIV and schistosomiasis services for fishermen, particularly investigating the effect of using social networks, HIV self-test kits and beach clinic services.

The specific objectives for the project are:

1. To conduct formative research to establish baseline data, identify peer- and sexual- networks in the intended study setting, optimise training and educational materials, and optimise HIV and praziquantel service delivery models.
2. To conduct a 3-arm cluster randomized trial (CRT) with a “boat-team” as the unit of randomization, comparing health and knowledge outcomes when outreach Beach Clinic services are promoted by peer educators (PE), or peer-HIVST distributor-educators (PDE), or standard beachside approaches. The primary outcomes will be
   1. uptake of either VMMC or ART during the intervention, and
   2. active *S. haematobium* infection (urine-positive for eggs).
3. To undertake within-trial economic evaluation to estimate costs and cost-effectiveness.
4. Investigate the role of social networks in delivery of trial services and effectiveness.

## **Methods**

### Study design and setting

This proposal is for a 3-arm project including formative work and a cluster randomised trial (CRT) using a “boat team” as the unit of randomisation. The intervention arms will compare 3 strategies (Figure 2) for creating demand for services among fishermen in Mangochi district. This will test the hypothesis that HIV self-testing (HIVST) promoted by peer educators (PE) or provided by peer-distributor-educators (PDE) will achieve high coverage of health interventions (recent HIV testing, linkage to VMMC and ART as indicated, and praziquantel) than beach-side services alone, and will leave men with increased understanding of the benefits of early treatment and prevention for both diseases.

**Figure 2:** Proposed trial summary


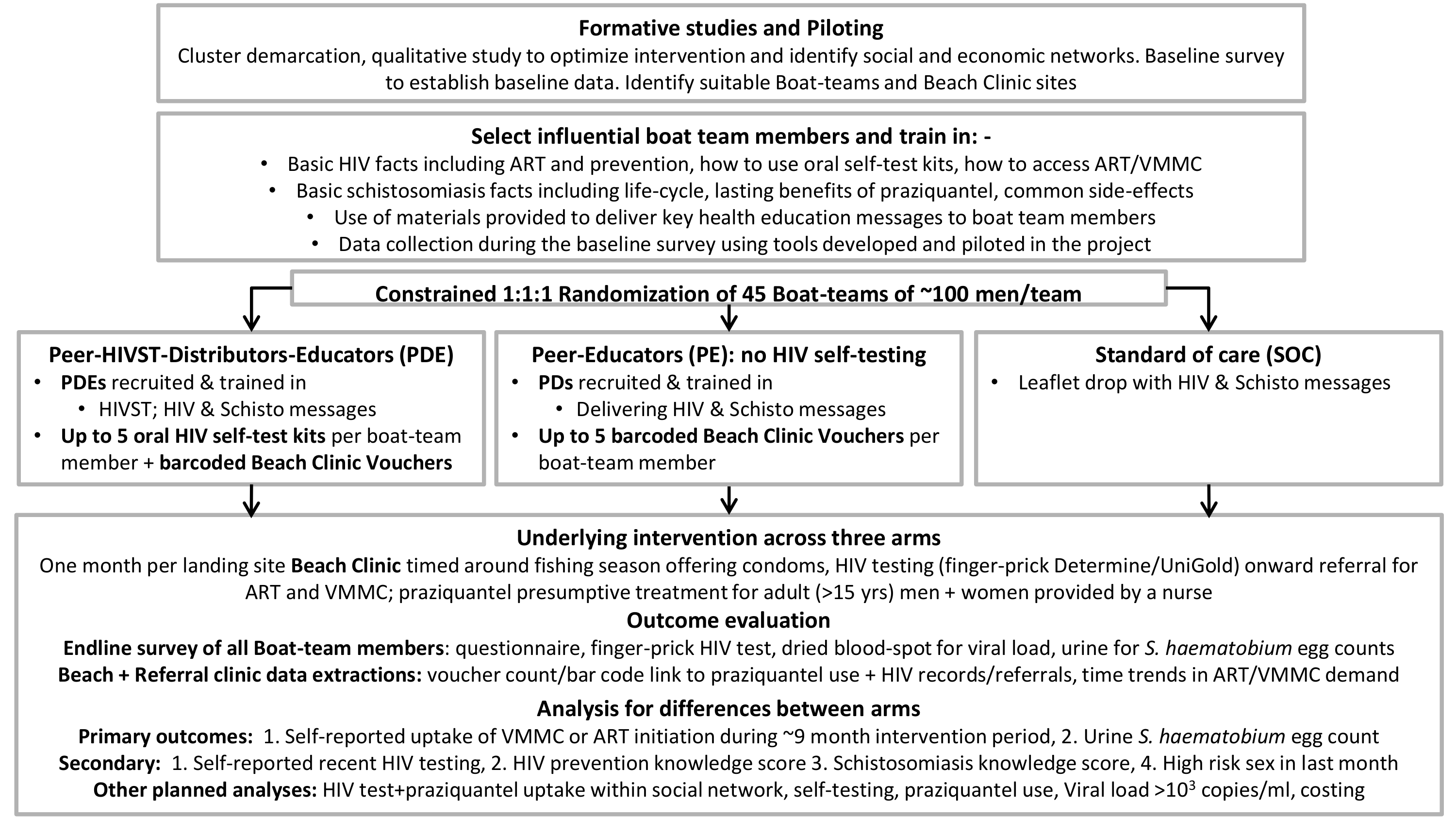


**Terms & Abbreviations:**

**ART**: antiretroviral therapy; **Boat team:** several boats and their cooperative; **PDE = Peer-distributor-educators** (study-recruited boat team volunteers briefly trained to provide peers with HIV self-test kits, information on HIV and schistosomiasis, and to promote Beach Clinic attendance); **PE = Peer educators**: study-recruited boat team volunteers briefly trained to provide peers information on HIV and schistosomiasis, and to promote Beach Clinic attendance; **SOC = Standard of care:** promotion of beach clinic services through usual village approach, with no formal peer-based system; **VMMC= voluntary medical male circumcision**

### Study population

The target population is fishermen aged 18 and over. This group has been traditionally missed during routine HIV and schistosomiasis services by the national programmes due to the nature of their occupation**.**

### Baseline and endline surveys

### Mapping, baseline survey and enumeration

A “boat team” is defined as a landing site or part of land from which fishermen dock. The initial activities will involve mapping these landing sites (**F01: Mapping form**) through circumferential walk with GPS devices. The aim will be to demarcate landing sites comprising ~100 fishermen (Figure 2). The exercise will also create “buffer” zones i.e. sufficient gaps between any two landing sites to avoid contamination during trial delivery. Potential locations for beach clinics will also be identified and marked during this exercise.

Following and during the mapping exercise, a survey will be conducted through selected potential “peer leaders” from each landing site (Figure 3). The peer leaders will administer the questionnaire to their fellow boat team members. The questionnaire will only include socio-demographic questions, some schistosomiasis-related questions and questions around willingness to attend the beach clinic (**F03: Baseline questionnaire).**

**Figure 3:** Project flow


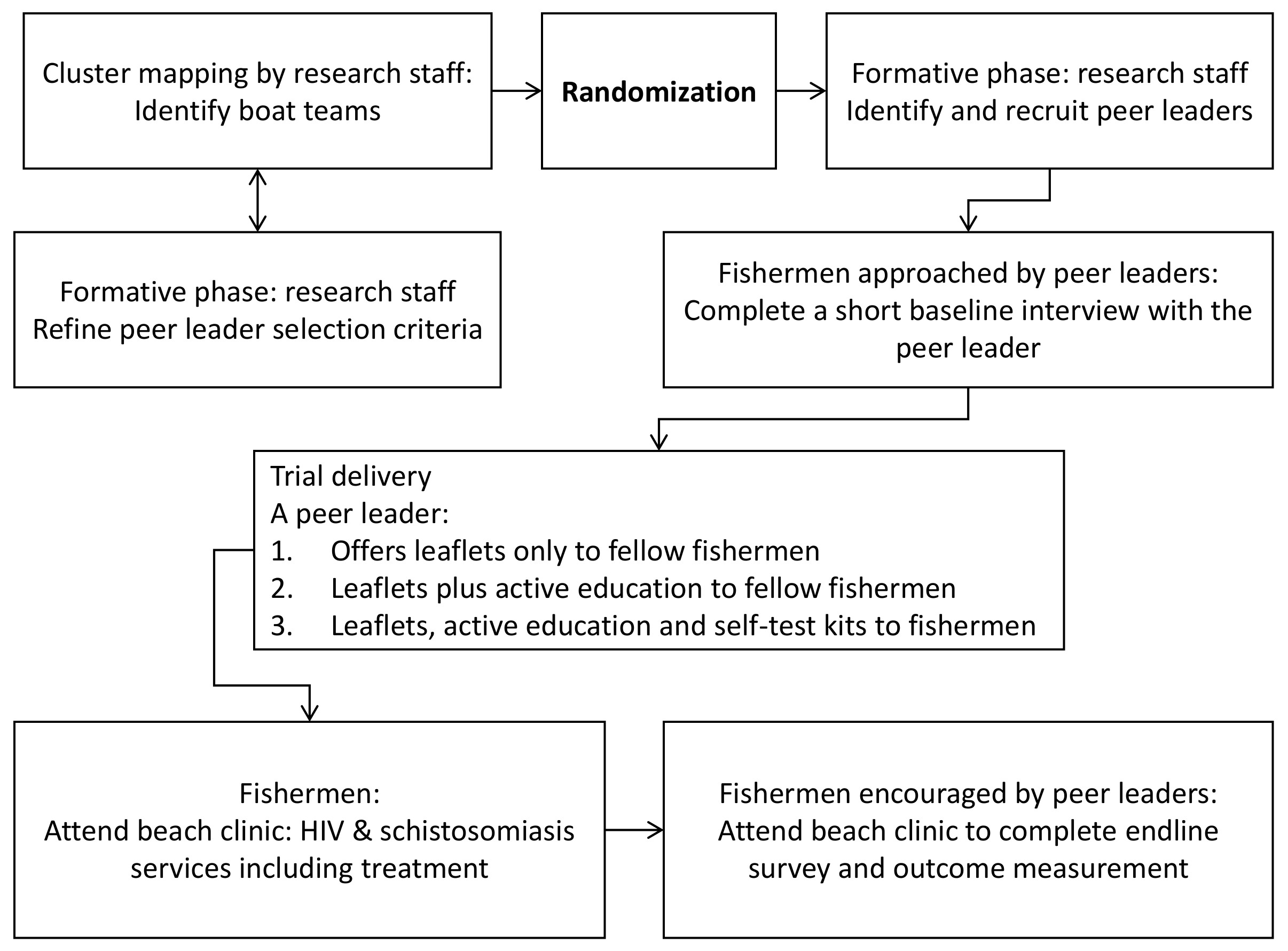


### Endline survey and data extraction

28 days after the last round of beach clinic services, an endline survey will follow up all consenting boat-team members (~4,500 individuals) through the peer leaders. Fishermen will be requested to attend the beach clinic for a face-to-face interview (**F13: Endline questionnaire)**, finger prick blood for immediate HIV testing, and a urine sample for egg-microscopy (**F14)**.

Data will be extracted from the Beach Clinic registers, using barcode readers to allocate trial arm origin of each participant.

### Randomisation and blinding

### Randomisation

Boat teams will be randomised 1:1:1 using computerized restricted randomization (geographical spread, cluster size, traditional authority, and HIV and schistosomiasis estimates) at a public randomisation ceremony. Investigator masking will be maintained for technical but not field staff or participants.

### Blinding

It is not practical to blind either the participants or the investigator in this study because of the nature of the interventions which include collection of different study materials such as HIV self-test kits. However, all data will be managed without reference to the study arm until the final data analysis, thus providing masking to main investigators and collaborators.

### Primary outcomes and measurement

Outcomes will compare differences between the two peer-based arms with SOC in costs and the following endpoints (Figure 2):

Primary outcomes measured at 9 months of trail delivery will compare differences between arms in the **proportions of boat-team fishermen**: -

1. Who self-report starting ART or undergoing VMMC during.
2. Who have ≥1 *S. haematobium* egg seen on light microscopy of the filtrate from 10mls urine (“egg-positive”).

### Secondary outcomes and measurement

Secondary outcomes will compare differences between arms in: -

1. Self-reported recent (last 9 months) HIV testing
2. Self-reported HIV prevention knowledge score
3. Self-reported schistosomiasis knowledge score
4. Self-reported high risk sex in the last month
5. *S. haematobium* intensity

Arms will also be compared for difference in self-reported praziquantel uptake and intensity of infection using egg count per 10mls of urine. Uptake of HIV and praziquantel in broader social networks will be investigated by constructing egocentric social networks.

### Trial procedures

### Trial implementation

Trial delivery will be through a phased approach implemented over a 9-month period with two beach clinics set up to cater for 5 clusters/boat teams per round (Figure 4). Each round will last one month before the next five clusters are activated.

**Figure 4:** Trial delivery schema for 45 clusters


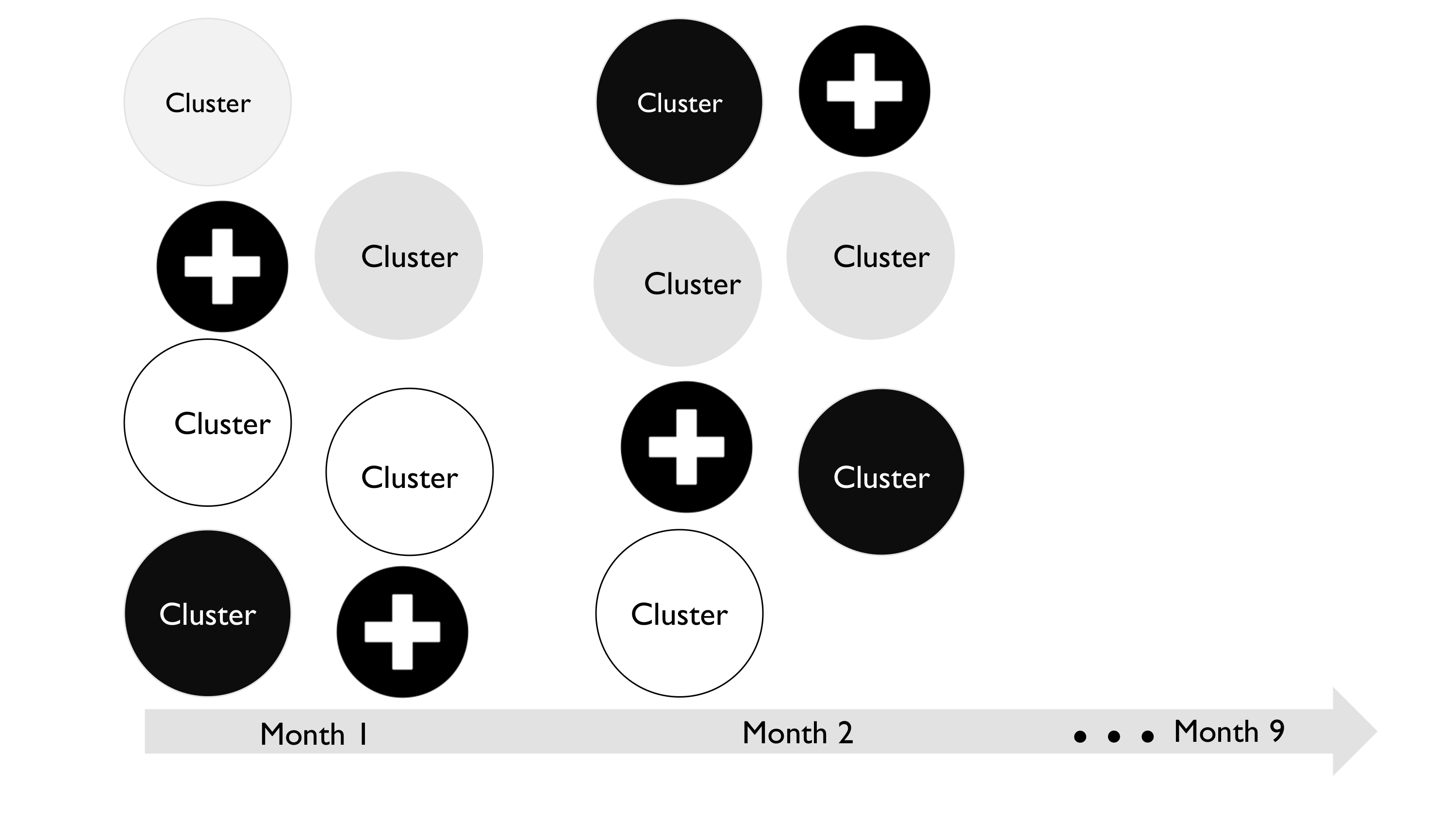


### Recruitment and training of peer-distributors/educators (PDEs and PEs)

For boat-teams randomised to peer-intervention arms, 2 to 5 PDE/PEs will be peer-nominated per boat-team for training by the study in basic HIV and schistosomiasis facts, and in how to pass on key messages to others using educational materials provided. PDEs will be trained in HIVST for secondary distribution. PDE/PEs will receive US$20 on meeting agreed intervention targets.

PEs will receive HIV/schistosomiasis educational and promotion materials and barcoded coloured vouchers to distribute to Boat-team members (5 vouchers per client) promoting Beach Clinic service uptake. Voucher recipients will be recorded in a register **(F07: Beach clinic attendance log/register).**

PDEs will receive educational materials as above and a starter supply of 50 HIVST kits for distribution, recorded in an HIVST **register (F06: linkage coupon for beach clinic attendance)** and allowing up to 5 HIVST kits per boat-team and 5 additional kits for wide community members (self, named friends and sexual partners) with reconciliation of register records whenever HIVST kits are restocked.

### Serious adverse events

All adverse events temporally related to participation in each trial arm particularly following delivery of HIV self-test kits and praziquantel will be captured according to a standard operating procedure for handling adverse events. The trial will only use the *grading* of adverse events: grade 1 (*mild), grade 2 (moderate), grade 3 (severe),* and *grade 4 (potentially life threatening) or grade 5 (death) in* order to classify all adverse events. Although all adverse events will be recorded, only grade 3-5 events, which are considered as serious adverse events (SAEs) will be reported to the institutional review boards (IRBs). Definitions for SAEs in relation to praziquantel will be standard ones as per drugs whereas for HIV self-testing the definitions below will be used:

| Definition of serious adverse event |
| --- |
| Any *grade 3, 4* or *5* events that occur within 30 days of delivering self-test kits to women or men enrolled in the trial.  Grade 3 events:   1. Intimate partner violence that leads to pain, bruising or marks within 24hrs. 2. Life-threatening violence (e.g. statement of intent to kill, strangulation, threatened with a knife or gun. 3. Physically coercive sex. 4. Reports fearing for his life. 5. Marriage break-up.   Grade 4 events:   1. Intimate partner violence leading to hospitalisation or death. 2. Suicide or attempted suicide. 3. Attack using potentially lethal force (e.g. knife, gun, hammer, and kicks to the head).   Grade 5:  1. Death |

Subsequently, a trial steering committee composed of investigators and members from the Ministry of Health will review all serious adverse events.

We will provide a range of options for supporting participants or peer educators who report any adverse events including: counselling offered by the study staff, referral to specialized counselling units for victims of violence, offering referral and/or transport to the hospital, and reporting to the local police, accordingly. This approach will be followed including for Grade 2 events that escalate over time into Grade 3, 4 or 5 events.

*Praziquantel side effects*

Common side effects for praziquantel are headache, nausea, abdominal pain, dizziness, drowsiness, fatigue, weakness, joint/muscle pains, loss of appetite, vomiting, sweating, itching. These are usually mild and transient, lasting from 30 minutes to up to 4 hours, which may be symptoms of the high intensity parasitic infection and or dying parasites.

We will manage these by doing the following:

1. Symptomatic treatment, reassurance and encouraging adequate fluids and rest.
2. Advising participants to take praziquantel after evening meal and as they go to bed, reduces the possibility of experiencing the side effects.

Serious side effects which are very rare include bloody diarrhoea, fever, irregular or slow heart beat or seizures/convulsions. Once these are experienced, we will refer the participant to the nearest health facility for management including supporting them with transportation. Thereafter, we will counsel the participant on schistosomiasis prevention and have alternative treatment whenever he has schistosomiasis.

### Beach clinics and laboratory methods

Beach clinic services will be provided from two tents located strategically to cover five clusters per round. Each beach clinic will be manned by experienced staff: one nurse-preferably seconded from Mangochi District Hospital; one HIV diagnostic assistant and one laboratory microscopist.

Egg count/10 ml will use filtration of a single urine specimen though Nuclepore (Whatman International Limited, Maidstone, England) membranes^10^, with 10% re-reading by a second reader. To estimate sampling error, a random 10% participants will have three specimens collected in late morning-early afternoon on three consecutive days, with serum stored for soluble egg antigen assay. These data will not contribute to the primary endpoint.

HIV testing will use OraQuick HIVST (an oral kit) or finger-prick testing with Determine and UniGold (**F15**).

### Sample size considerations

Using established cluster-randomised methodology^32^ 15 boat-teams/arm (1,500 fishermen/arm) will provide 80% power to detect a 9% increase in combined ART/VMMC uptake compared to an assumed 10% under SOC^20^. We assume intercluster coefficient of variation (*k*) of 0.20, with HIVST uptake of 50%-80%^20,23^ (Table 1).

For the **second primary** outcome, we assume praziquantel uptake will be 10% to 20% higher in PDE/PE arms than an assumed 40-60% for the SOC arm**,** with baseline egg-positivity 15-25%, 95% cure from praziquantel, and *k* 0.20-0.30.^10,33^ Power provided by the 15 clusters/arm for HIV endpoints is >80% over most of this range of scenarios.

**Table 1:** Assumptions and parameters for sample size

| **Assumptions** | |
| --- | --- |
| Average cluster size (number of men in a fishing dock) | 100 |
| Proportion eligible for the trial (mainly not already on ART) | 0.80 |
| Proportion accepting to self-test | 0.50 – 0.80^20,23^ |
| Proportion HIV positive or HIV negative and uncircumcised | 0.20 – 0.50^20^ |
| Uptake of praziquantel in SOC arm by endline | 0.4-0.6 |
| Proportion urine egg-positive in SOC arm (endline) | 0.09-0.17 |
| Geometric mean egg-count if egg-positive in SOC (endline) | 10/10mL |
| **Parameters** | |
| Significance level (α) | 0.05 |
| Power (1-β) | 0.80 |
| Allocation ratio | 1:1:1 |

### Risk mitigation and contingency

All intervention components are already National Policy. HIVST social harms and praziquantel side effects will be reported to ethics, TSC, DSMB only if serious (Grade 3 or 4) consistent with international recommendations for post-marketing trials, with passive reporting and management of social harms during HIVST using established methods^21^.

## **Sub-studies**

### Economic evaluation

An Economic analysis will be undertaken to estimate the costs and cost-effectiveness of the interventions. Primary costing will be utilised to estimate the costs of providing the Beach Clinic services (SOC), and the interventions being provided in the two intervention arms. Resources utilised will be captured by review of trial expenditure reports. Total estimated costs will be used in conjunction with observed primary outcomes to estimate cost per individuals tested for HIV and linked to VMMC/ART services, and cost per individual treated for schistosomiasis. An incremental analysis will estimate the additional costs per additional individual tested for HIV and linked to VMMC/ART services, and per additional individual treated for schistosomiasis. The incremental analysis will compare the two intervention arms to current SOC. The health economics component of the fellowship will inform whether the additional resources utilised in utilizing peers to educate and/or distribute HIVST offers value for money in Malawi.

### Social science

The main research question to be addressed during this qualitative study will be:

What is the composition, hierarchy and inter-/intra-boat team relations/interactions as a social or economic network? Specific objectives for the formative component will include:

1. To explore the design, social composition and membership of the fishing boat teams.
2. To understand social activities, social relations/networks and patterned interactions within and between boat teams.
3. To examine the hierarchy of authority and characterize attributes within and between boat teams to identify boat team members who potentially influence the behavior of other members (peer leaders). The peer leaders will later act as agents for trial delivery.
4. To characterise attributes of peer leaders and develop criteria for identifying and selecting individual members of boat teams to become peer leaders.
5. To define *social influence* as a concept for optimizing delivery of health interventions.

A qualitative research design will be employed to understand the composition, hierarchy and inter-boat team interaction as a social or economic network. This formative qualitative sub-study will inform the design and shape the development and implementation processes of the trial. A total of 7 focus group discussions (FGD) and one participatory workshop are planned for this phase with consenting and purposively identified fishermen at a convenient location. Participants in FGDs will be fishermen identified during the mapping exercise as potential peer leaders or peer educators by study field workers. Contact details will be collected during the mapping exercise followed by verbal invitation to participate in the FGD.

Pre-developed and piloted semi-structured interview guide will guide the discussion. Data on boat-team size/mobility, optimum service configuration of Beach Clinics, barriers to uptake of VMMC and ART (**F02a & F02b: FGD question guides)**. We will also test proposed intervention materials and solicit concerns relating to HIVST or praziquantel. A workshop manual - containing clearly defined participatory activities and outcome for each activity - will guide activities within a stakeholder participatory workshop. Data collection will be led by a Post-doctoral social scientist, and with support from an experienced junior social science researcher.

Group discussions and the stakeholder participatory workshop will be conducted in Chichewa (a local language) and will be recorded. Recorded data will be transcribed verbatim, translated and uploaded onto NVIVO 12 software for data management and coding. Thematic qualitative data analysis will be used to analyse and interpret data.

### Social networks

The aim of this component is to assess the effect of using egocentric social networks to increase uptake of HIV and schistosomiasis services at the beach clinic. We assume that the peer leaders will be identified during the social science and the baseline survey phase will be able to influence both their fellow boat team members and the wider community. Thus, they will be designated as egos with their fellow boat team members and the wider community members as the alteri (Figure 5). Here, we will aim to address the following specific objectives:

1. To understand the size of the egocentric social networks.
2. To assess the composition of the egocentric social networks: homophilly and heterogeneity.
3. To examine the structure of the egocentric social networks including the degree, strength of ties and the average strength of ties.

In order to implement this component, we will use different colour coded vouchers given to the egos at distribution point to differentiate between fishermen and wider community members. Implementation of this approach will be in all three arms with specific methods of measuring spillover effects employed at analysis stage. These vouchers will then be tracked at the beach clinic with documentation in **F07:** Beach clinic attendance log/register with respect to the type of participant who visited the beach clinic.

**Figure 5:** Schema of egocentric social network


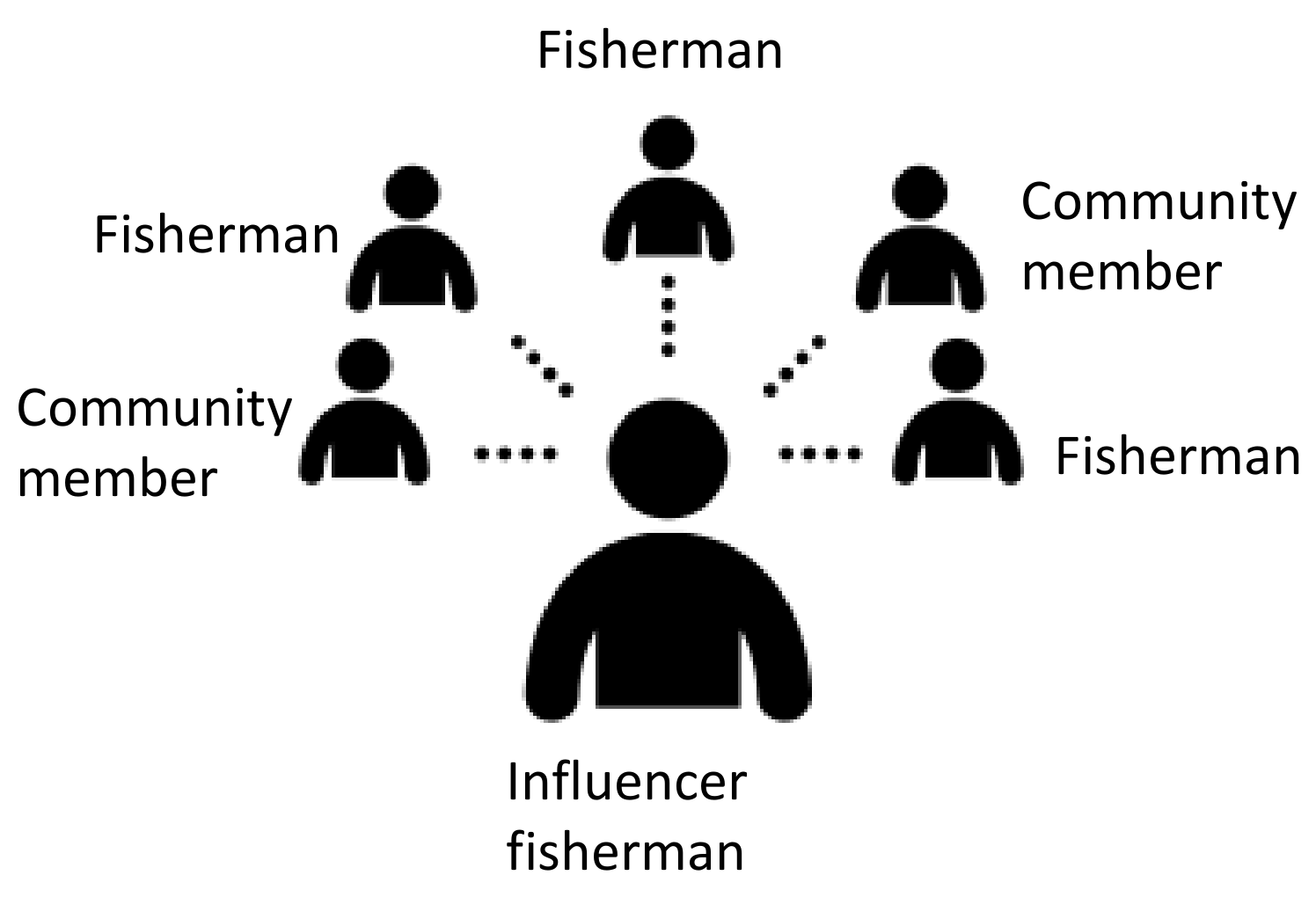


## **Timelines**

**Figure 5:** project timelines

## **Data management and statistical analysis**

### Data management

Data will be managed through infrastructure set up within Malawi-Liverpool-Wellcome Trust Clinical Research Programme (MLW). Data collection and processing will be as detailed in the data management plan (DMP) developed for the project. Data will be collected using tablets running Open Data Kit (ODK) and will be downloaded onto a server running a MySQL Relational Database. A mirror paper-based system will be set up as backup for troubleshooting in case of device or software failure.

Data quality assurance will be implemented within the electronic form so that out of range values, inconsistent values and required variables will be checked at the time of data collection. All tablets will have full log-in details of the person collecting the data including a password. Access to the study database will be protected by a password known only to the PI (Augustine Choko) and the IT systems administrator in MLW. Data for study monitoring will be periodically exported into comma separated values (CSV) from the study database on the MLW server for analysis and to raise plus resolve data queries.

Protocols for managing data without breach of confidentiality are in place within MLW. Access to the final data set will be limited to the PI (Augustine Choko), co-principal investigators and colleagues at the HIV/AIDS Department and the schistosomiasis Control Programme listed as co-investigators. Sensitive information (including HIV results) will not be linked to personal identifiers in the final data set.

All devices and paper-based tools containing data will be kept in locked offices at MLW during data processing and in a locked data repository room for longer term storage. All data will be backed up daily by the MLW Data Office, with offsite back up once weekly. Backup data will be stored in a locked filing cabinet away from the office by the PI.

### Statistical analysis

Trial analyses will use R^34^ and Stata 14.0. Baseline and endline characteristics will be computed as proportions or median (interquartile range [IQR]), or negative binomial (eggs/10ml), as appropriate, by arm. Imbalances will be adjusted for in primary and secondary outcomes. All analyses will be intention-to-treat with adjustment for clustering. A full statistical plan will be written before unblinding.

## **Piloting**

The training curriculum and educational materials for the two peer-based arms will be developed from existing materials (<http://hivstar.lshtm.ac.uk/protocols/>). Materials explaining key aspects of schistosomiasis and benefits of presumptive treatment will be adapted from control programme materials.

The two peer-based intervention arms will be piloted in a single community outside the trial sites. Boat-teams will be randomly allocated to one of the peer-based arms (no standard of care) and asked to nominate 2 to 5 peers for 1.5 days training.

Process evaluation plans and endline survey tools will be developed and piloted.

Once finalized, the planned intervention materials, Beach Clinic locations, and timing (start and finish of the fishing season: March and November) will be agreed with Traditional Authorities, District Health Office, and Ministry of Health.

## **Ethics, conflict of interest, data availability and dissemination**

### Ethical approval

Ethics approval will be sought from Ethics Committee of LSTM and Malawi College of Medicine (COMREC). A trial steering committee, data safety monitoring board and community advisory board will be established. New HIV patients will be counselled and referred for treatment. Participants with active schistosomiasis will be treated. Written or thumb print informed consent will be taken from all participants, except for the intervention where we will request waiver of informed consent given the nature of the intervention (Table 2). Leaflets (L01a/L01b) and educational materials will clearly state the investigational nature and provide a telephone number and information on locally available HIV clinic services.

**Table 2:** Consent requirements for research activities

| **Research activity** | **Research component** | **Consent requirements** |
| --- | --- | --- |
| Mapping of clusters (landing sites) | Cluster definition | Consent not required but permission from local leaders |
| Focus group discussions | Formative study | Written (witnessed) informed consent |
| Baseline survey | Listing and baseline data collection | Written (witnessed) informed consent |
| HIVST kit provision for secondary distribution | Intervention | Request waiver of informed consent |
| HIVST kit recipient | Intervention | Request waiver of informed consent (leaflet provided in lieu) |
| Endline survey including collection of samples | Outcomes evaluation | Written (witnessed) informed consent |
| Routine clinic data extraction | Outcome evaluation | Not applicable |
| Direct observation | Health economics | No consent required |
| Informal interviews | Health economics | Verbal |
| Extraction of programme and government expenditure reports, databases and logs | Health economics | Verbal |

Waiver of informed consent for individuals providing and accepting HIVST kits is requested on the following grounds:

- The intervention is being supported by MoH and is national policy^25^.
- HIVST is now recommended as international best practice by WHO ^35^
- The OraQuick HIV Self-Test has been evaluated and approved by WHO Prequalification ^36^.
- Secondary-distribution is becoming an established practice, with minimal risk of harms as demonstrated in Malawi and other sub-Saharan African countries ^30^.
- The trial aims to assess a pragmatic, unrestricted intervention, with potential integration and scale-up by MoH. Formal consent procedures would by definition affect the nature of the intervention.

### Sponsor

Liverpool School of Tropical Medicine will act as the main sponsor for this study. Delegated responsibilities will be assigned locally.

### Conflict of interest

Neither the PI nor any of the collaborators has any conflict of interest.

### Compensation for participants

Participants in the qualitative component will be compensated K2000 for 1-hour time and transport to the venue of the discussion. Endline survey participants will receive K4000 for 1-hour time and 2 mild procedures (blood and urine sample collection). There will be no compensation for participation in the baseline survey because selected peers will complete a short (5 minutes) interview at the location of fishing. Similarly, there will be no compensation for accepting any services during trial delivery as doing so could amount to inducement and to ensure sustainability of the model in the hands of the MoH. The national guidelines for HIV or schistosomiasis do not require patients to be paid to access services. Thus, giving compensation for accessing these services at a location conveniently located to participants would go against the national guidelines and would set a dangerous precedence of expectations in the community about clinic attendance.

### Data availability

The final fully anonymised data from the study will be made publicly available through the LSHTM data repository (<http://datacompass.lshtm.ac.uk/>.

### Dissemination

Findings from the trial will be primarily aimed to inform the Ministry of Health (MoH) through presentations and final copy of the report. Further local dissemination will be done at the National AIDS Commission (NAC) / College of Medicine (COM) annual dissemination conference. Findings will also be presented at peer-reviewed regional and international conferences. Copies of the final report, published peer-reviewed paper (s) and abstracts will be made available to the COM Library, and to College of Medicine Research Ethics Committee (COMREC).

## **Training and capacity building**

This work is anticipated to be part of my (Augustine Choko) Post-Doctoral training with London School of Hygiene & Tropical Medicine. This Fellowship will advance my skills in statistical and social networks (six months at John Hopkins University with Prof Stephane Helleringer) and advanced statistical modelling and R programming (three months at LSHTM). Study personnel including selected fishermen will be trained on research ethics, maintaining confidentiality and data collection using tablets among other essential skills. All study staff including the principal investigator (PI) will undergo Good Clinical Practice (GCP) training or refresher GCP training as appropriate.

## **Personnel, materials and consumables, equipment, space**

### Personnel

Peer fishermen who will be identified in the early phase (social science) of the study will be responsible for participant recruitment and baseline data capture. HIV testing services will be conducted by HIV counsellors who will be hired as part of the study. Schistosomiasis microscopy will be conducted by microscopists whereas a study nurse seconded from the nearest health center in the study site will provide praziquantel. The HIV counsellors, the microscopists and the nurse will run the beach clinic in a tent and will manage follow-up interviews. A study coordinator will supervise the peer fishermen while the PI will provide overall leadership of the study. A medical doctor who is a co-investigator will provide clinical oversight particularly regarding the any side effects from praziquantel.

### Materials and consumables

The following materials and consumables will be required for the study

1. OraQuick ADVANCE HIV I/II (OraSure Technologies, Bethlehem, USA) for oral tests
2. Determine 1/2™ (Alere, Waltham, USA) and Uni-Gold™ Recombigen^®^ HIV (Trinity Biotech, Bray, Ireland) for confirmatory HIV testing using finger prick blood.
3. Urine containers, preferably 60 mls volume
4. Filter holders with gaskets
5. Nuclopore membranes for urine filtration
6. 20ml syringes without needles
7. Writing materials, study materials, visual aids and clip boards will be required during protocol training and other training activities.
8. Printing
9. Vehicle running costs

### Equipment

Tablets for electronic data capture, lockable filing cabinets for temporary storage of completed consent forms and study tools. Light microscope with an electricity power supply to be used for examination of the filters after urine filtration.

### Space

Space for storing study tools, equipment and consumables as well as private space for conducting study procedures will be required in all recruitment primary health centers.

### Miscellaneous

Airtime for mobile communication between study personnel will be required.

## **Budgetary estimate**

**Table 3:** Budget

## **References**

1. Joint United Nations Programme on HIV and AIDS (UNAIDS). UNAIDS Data. Geneva, Switzerland: UNAIDS, 2018.

2. Malawi Ministry of Health. Malawi Population-based HIV Impact Assessment (MPHIA) 2015-16: First Report. Lilongwe, Malawi, 2017.

3. Joint United Nations Programme on HIV and AIDS (UNAIDS). Ending AIDS. Progress towards the 90–90–90 targets. Geneva, Switzerland: UNAIDS, 2017.

4. MacPherson EE, Sadalaki J, Njoloma M, Nyongopa V, Nkhwazi L, Mwapasa V, Lalloo DG, Desmond N, Seeley J, Theobald S. Transactional sex and HIV: understanding the gendered structural drivers of HIV in fishing communities in Southern Malawi. *Journal of the International AIDS Society* 2012; **15 Suppl 1**: 1-9.

5. Cohen MS, Chen YQ, McCauley M, Gamble T, Hosseinipour MC, Kumarasamy N, Hakim JG, Kumwenda J, Grinsztejn B, Pilotto JH, Godbole SV, Chariyalertsak S, Santos BR, Mayer KH, Hoffman IF, Eshleman SH, Piwowar-Manning E, Cottle L, Zhang XC, Makhema J, Mills LA, Panchia R, Faesen S, Eron J, Gallant J, Havlir D, Swindells S, Elharrar V, Burns D, Taha TE, Nielsen-Saines K, Celentano DD, Essex M, Hudelson SE, Redd AD, Fleming TR. Antiretroviral Therapy for the Prevention of HIV-1 Transmission. *The New England journal of medicine* 2016; **375**(9): 830-9.

6. Rodger AJ, Cambiano V, Bruun T, Vernazza P, Collins S, van Lunzen J, Corbelli GM, Estrada V, Geretti AM, Beloukas A, Asboe D, Viciana P, Gutierrez F, Clotet B, Pradier C, Gerstoft J, Weber R, Westling K, Wandeler G, Prins JM, Rieger A, Stoeckle M, Kummerle T, Bini T, Ammassari A, Gilson R, Krznaric I, Ristola M, Zangerle R, Handberg P, Antela A, Allan S, Phillips AN, Lundgren J. Sexual Activity Without Condoms and Risk of HIV Transmission in Serodifferent Couples When the HIV-Positive Partner Is Using Suppressive Antiretroviral Therapy. *Jama* 2016; **316**(2): 171-81.

7. Kayuni S, Lampiao F, Makaula P, Juziwelo L, Lacourse EJ, Reinhard-Rupp J, Leutscher PDC, Stothard JR. A systematic review with epidemiological update of male genital schistosomiasis (MGS): A call for integrated case management across the health system in sub-Saharan Africa. *Parasite epidemiology and control* 2019; **4**: e00077.

8. Wall KM, Kilembe W, Vwalika B, Dinh C, Livingston P, Lee YM, Lakhi S, Boeras D, Naw HK, Brill I, Chomba E, Sharkey T, Parker R, Shutes E, Tichacek A, Secor WE, Allen S. Schistosomiasis is associated with incident HIV transmission and death in Zambia. *PLoS neglected tropical diseases* 2018; **12**(12): e0006902.

9. World Health Organization. Accelerating work to overcome the global impact of neglected tropical diseases: a roadmap for implementation. Geneva, Switzerland: WHO, 2011.

10. Kayuni S, Peeling R, Makaula P. Prevalence and distribution of Schistosoma haematobium infection among school children living in southwestern shores of Lake Malawi. *Malawi medical journal : the journal of Medical Association of Malawi* 2017; **29**(1): 16-23.

11. Toor J, Alsallaq R, Truscott JE, Turner HC, Werkman M, Gurarie D, King CH, Anderson RM. Are We on Our Way to Achieving the 2020 Goals for Schistosomiasis Morbidity Control Using Current World Health Organization Guidelines? *Clinical infectious diseases : an official publication of the Infectious Diseases Society of America* 2018; **66**(suppl_4): S245-s52.

12. Mwanakasale V, Vounatsou P, Sukwa TY, Ziba M, Ernest A, Tanner M. Interactions between Schistosoma haematobium and human immunodeficiency virus type 1: the effects of coinfection on treatment outcomes in rural Zambia. *The American journal of tropical medicine and hygiene* 2003; **69**(4): 420-8.

13. National Statistical Office (NSO) [Malawi] and ICF. Malawi Demographic and Health Survey 2015-16. Zomba, Malawi, and Rockville, Maryland, USA. NSO and ICF: Zomba, Malawi, and Rockville, Maryland, USA. NSO and ICF, 2017.

14. Skovdal M, Campbell C, Madanhire C, Mupambireyi Z, Nyamukapa C, Gregson S. Masculinity as a barrier to men's use of HIV services in Zimbabwe. *Global Health* 2011; **7**(1): 13.

15. Siu GE, Wight D, Seeley JA. Masculinity, social context and HIV testing: an ethnographic study of men in Busia district, rural eastern Uganda. *BMC Public Health* 2014; **14**(1): 33.

16. Siu GE, Wight D, Seeley J. 'Dented' and 'resuscitated' masculinities: the impact of HIV diagnosis and/or enrolment on antiretroviral treatment on masculine identities in rural eastern Uganda. *SAHARA J* 2014; **11**(1): 211-21.

17. Izugbara CO, Undie C-C, Mudege NN, Ezeh AC. Male youth and Voluntary Counseling and HIV-Testing: the case of Malawi and Uganda. *Sex Education* 2009; **9**(3): 243-59.

18. Chikovore J, Gillespie N, McGrath N, Orne-Gliemann J, Zuma T, Group ATS. Men, masculinity, and engagement with treatment as prevention in KwaZulu-Natal, South Africa. *AIDS Care* 2016; **28 Suppl 3**: 74-82.

19. Chikovore J, Hart G, Kumwenda M, Chipungu GA, Corbett L. 'For a mere cough, men must just chew Conjex, gain strength, and continue working': the provider construction and tuberculosis care-seeking implications in Blantyre, Malawi. *Glob Health Action* 2015; **8**(1): 26292.

20. Choko AT, Corbett EL, Stallard N, Maheswaran H, Lepine A, Johnson CC, Sakala D, Kalua T, Kumwenda M, Hayes R, Fielding K. HIV self-testing alone or with additional interventions, including financial incentives, and linkage to care or prevention among male partners of antenatal care clinic attendees in Malawi: An adaptive multi-arm, multi-stage cluster randomised trial. *PLoS medicine* 2019; **16**(1): e1002719.

21. Choko AT, Fielding K, Stallard N, Maheswaran H, Lepine A, Desmond N, Kumwenda MK, Corbett EL. Investigating interventions to increase uptake of HIV testing and linkage into care or prevention for male partners of pregnant women in antenatal clinics in Blantyre, Malawi: study protocol for a cluster randomised trial. *Trials* 2017; **18**(1): 349.

22. Choko AT, Kumwenda MK, Johnson CC, Sakala DW, Chikalipo MC, Fielding K, Chikovore J, Desmond N, Corbett EL. Acceptability of woman-delivered HIV self-testing to the male partner, and additional interventions: a qualitative study of antenatal care participants in Malawi. *Journal of the International AIDS Society* 2017; **20**(1): 21610.

23. Choko AT, Nanfuka M, Birungi J, Taasi G, Kisembo P, Helleringer S. A pilot trial of the peer-based distribution of HIV self-test kits among fishermen in Bulisa, Uganda. *PLoS One* 2018; **13**(11): e0208191.

24. The CDI Study Group. Community-directed interventions for priority health problems in Africa: results of a multicountry study. *Bulletin of the World Health Organization* 2010; **88**(7): 509-18.

25. Malawi Ministry of Health. Malawi HIV Self-Testing Operational Guidelines. Lilongwe, Malawi, 2018.

26. MacPherson P, Lalloo DG, Webb EL, Maheswaran H, Choko AT, Makombe SD, Butterworth AE, van Oosterhout JJ, Desmond N, Thindwa D, Squire SB, Hayes RJ, Corbett EL. Effect of optional home initiation of HIV care following HIV self-testing on antiretroviral therapy initiation among adults in Malawi: a randomized clinical trial. *Jama* 2014; **312**(4): 372-9.

27. Shelton RC, Lee M, Brotzman LE, Crookes DM, Jandorf L, Erwin D, Gage-Bouchard EA. Use of social network analysis in the development, dissemination, implementation, and sustainability of health behavior interventions for adults: A systematic review. *Social science & medicine (1982)* 2019; **220**: 81-101.

28. Peters PJ, Gay C, Beagle S, Shankar A, Switzer WM, Hightow-Weidman LB. HIV infection among partners of HIV-infected black men who have sex with Men - North Carolina, 2011-2013. *MMWR Morbidity and mortality weekly report* 2014; **63**(5): 90-4.

29. Valente TW. Network interventions. *Science (New York, NY)* 2012; **337**(6090): 49-53.

30. WHO. Guidelines on HIV self-testing and partner notification: supplement to consolidated guidelines on HIV testing services. Geneva, Switzerland: World Health Organization (WHO), 2016.

31. Bustinduy A L, King C H. Schistosomiasis. In: Farrar J, Hotez P, Junghanss T, Lalloo D, White N, eds. Manson’s Tropical Diseases. 23 ed. London: Elsevier; 2014.

32. Hayes JR, Moulton LH. Cluster Randomised Trials: Chapman and Hall/CRC; 2009.

33. Chipeta MG, Ngwira B, Kazembe LN. Analysis of Schistosomiasis haematobium infection prevalence and intensity in Chikhwawa, Malawi: an application of a two part model. *PLoS neglected tropical diseases* 2013; **7**(3): e2131.

34. R Core team. R: A language and environment for statistical computing. R Foundation for Statistical Computing, Vienna, Austria. URL <http://www.R-project.org/>. 2015.

35. World Health Organization. Guidelines on HIV self-testing and partner notification. Supplement to consolidated guidelines on HIV testing services. Geneva, Switzerland: WHO, 2016.

36. WHO. WHO prequalification of in vitro diagnostics: OraQuick HIV Self-Test. Geneva, Switzerland: World Health Organization (WHO); 2017.

## **Appendices**

### Tools for FISH project

**Note** that these tools will be developed and stamped by COMREC in due course except for consent forms which have been included in this submission.

**Mapping**

**F01: FISH mapping form.** Form for demarcating clusters.

**Qualitative**

**F02a:** Peer leader selection focus group discussion guide

**F02b:** Focus group discussion guide

**C01:** Consent and information sheet for FGD participation

**F08:** Quantitative data for FGD participants

**Baseline survey**

**F03:** Baseline questionnaire

**C02:** Consent and information sheet for baseline interview

**Trial delivery**

**F04:** coupon distribution register

**F05:** Self-test distribution register

**F06:** Coupon for beach clinic attendance (linkage) (F06a and F06b, Eng and Chic)

**F07:** Beach clinic attendance log/register

**Endline survey**

**F09:** Beach clinic attendance log/register for endline interview

**F12:** Urine egg count form

**F13:** Endline questionnaire

### Potential landing sites (clusters) in Mangochi

| **FISH STUDY LANDING SITES** | | |  |
| --- | --- | --- | --- |
| **MH-BOMA LAKESIDE/LOCATION** | | |  |
| **No** | **SITE NAME** | **POPULATION** | **NEARBY ART CLINIC** |
| 1 | Bolera |  |  |
| 2 | Chimatilo |  |  |
| 3 | Namiyasi |  |  |
| 4 | Masanga |  |  |
| 5 | Nkungumbe |  |  |
| 6 | Nasenga |  |  |
| 7 | Chikundo |  |  |
| 8 | Chisigele |  |  |
| 9 | Mtimbuka |  |  |
| 10 | Michesi |  |  |
| 11 | Chipoka |  |  |
| 12 | Mpemba |  |  |
| 13 | Mwawa |  |  |
| 14 | Matuwi |  |  |
| 15 | Maudzu |  |  |
| 16 | Mpale |  |  |
| 17 | Mpeta |  |  |
| 18 | Chemdala |  |  |
| 19 | Chindongo |  |  |
| 20 | Nabale |  |  |
| 21 | Lizimba |  |  |
| 22 | Nkope |  |  |
| 23 | Kalenjeka |  |  |
| 24 | Khuzi |  |  |
| 25 | Namasobay |  |  |
| 26 | Nagoma |  |  |
| 27 | Kasanga |  |  |
| **CAPEMACLEAR LAKESIDE/LOCATION** | | |  |
| **No** | **SITE NAME** | **POPULATION** | **NEARBY ART** |
| 28 | Malembo |  |  |
| 29 | Msaka |  |  |
| 30 | Mvunguti |  |  |
| 31 | Nsangu |  |  |
| **MAKANJIRA LAKESIDE/LOCATION** | | |  |
| **No** | **SITE NAME** | **POPULATION** | **NEARBY ART** |
| 32 | Bala |  |  |
| 33 | Malindi fisheries |  |  |
| 34 | Katundu |  |  |
| 35 | Chikolomo/Pajangiya |  |  |
| 36 | Namnumbwa |  |  |
| 37 | Makumba |  |  |
| 38 | Mwala/Pamoyo |  |  |
| 39 | Chebuleki/Pachapola |  |  |
| 40 | Mpundi |  |  |
| 41 | Ng`ombe |  |  |
| 42 | Fowo |  |  |
| 43 | Machakwani/mdoka |  |  |
| 44 | Mbale |  |  |
| 45 | Namalaka |  |  |
| 46 | Kadango |  |  |
| 47 | Nyangu |  |  |
| 48 | Pabakili |  |  |
| 49 | Chemdala |  |  |
| 50 | Chenamtiosi |  |  |
| 52 | Mauni |  |  |
| 53 | Lulanga |  |  |
| 54 | Mpilipili |  |  |
| 55 | Chiphole |  |  |
| 56 | Kanjanje |  |  |
